# Supplementary material for: Isoform prefiltering improves performance of count-based methods for analysis of differential transcript usage
Source: Genome Biol. 2016 Jan 26;17:12. doi: 10.1186/s13059-015-0862-3 (PMC4729156; doi:10.1186/s13059-015-0862-3)
Supplement: Additional file 1 — Supplementary figures, tables, and methods. Additional file 1 contains supplementary figures and tables referred to in the text. It also contains a description of the counting methods and a comparison of the isoform-guided filtering with bin-level filtering. (PDF 9520 KB) [file 13059_2015_862_MOESM1_ESM.pdf]

Isoform prefiltering improves performance of count-based methods for analysis of differential transcript usage
Supplementary Material

Charlotte Soneson†, Katarina L. Matthes†, Malgorzata Nowicka,
Charity W. Law, Mark D. Robinson

Contents

1 Illustration of differences between counting bin definitions 3

2 Comparison of the two transcriptomes used for the simulations 5

3 Simulation and study design details 7

4 Additional information about the counting methods 11
4.1 MISO 11
4.2 DEXSeq 11
4.3 featureCounts 11
4.4 kallisto 11
4.5 SplicingGraphs 11
4.6 casper 12
4.7 TopHat junction counts 12

5 Comparison of count patterns 13

6 Evaluation of cuffdiff with a modified gtf file 16

7 Performance evaluation stratified by the type of alternative splicing event 18

8 Performance evaluation based on only genes shared between results and truth 19

9 Performance evaluation stratified by compositional difference between differentially used isoforms 20

10 rMATS FPs occur due to events with very low prevalence 21

11 Performance evaluation stratified by overall gene expression level 22

12 Performance evaluation stratified by incomplete annotation-supplementary figures 23

13 Characterisation of false positives found by *DEXSeq-noaggreg* 28

† Equal contribution

14 Improved FDR control with filtering **30**
14.1 *DEXSeq-noaggreg* 30
14.1.1 FPs differing between original and 5% filtered with DEXSeq-noaggreg 39
14.2 *kallisto* 46
14.3 *rMATS* 48

1 Illustration of differences between counting bin definitions

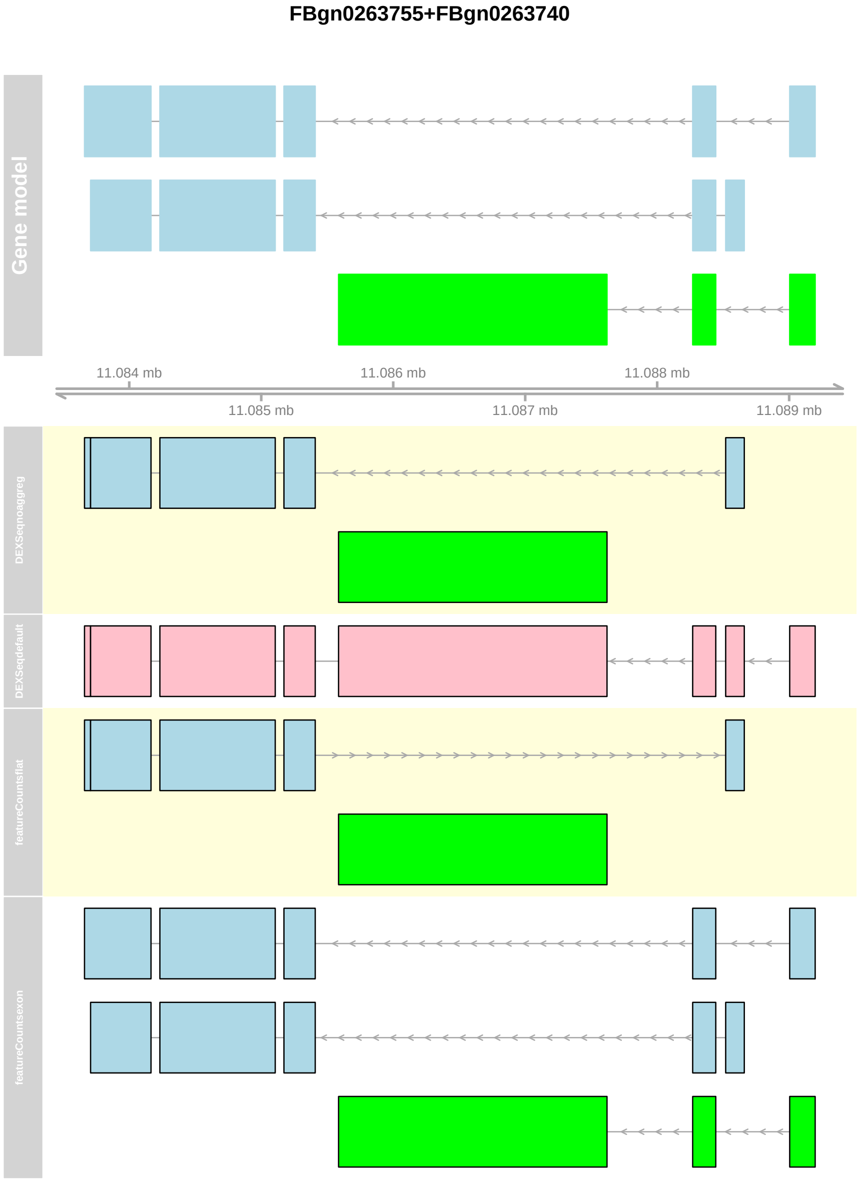

Supplementary Figure 1: Illustration of the differences between bins generated for *DEXSeq-noaggreg*, *DEXSeq-default*, *featureCounts-flat* and *featureCounts-exon*, for two overlapping genes on the same strand. The top panel shows the gene models for the overlapping genes (transcripts from the different genes are colored in different colors). The panels below the genome coordinate track show the counting bins defined by each method (from top to bottom: *DEXSeq-noaggreg*, *DEXSeq-default*, *featureCounts-flat* and *featureCounts-exon*). Bins assigned to different genes/complexes are colored in different colors. Here, *DEXSeq-default* merges the overlapping genes into a complex, while *DEXSeq-noaggreg* and *featureCounts-flat* instead exclude the overlapping parts and define bins based only on regions that are unique to one gene. *featureCounts-exon* uses the original exons as counting bins.

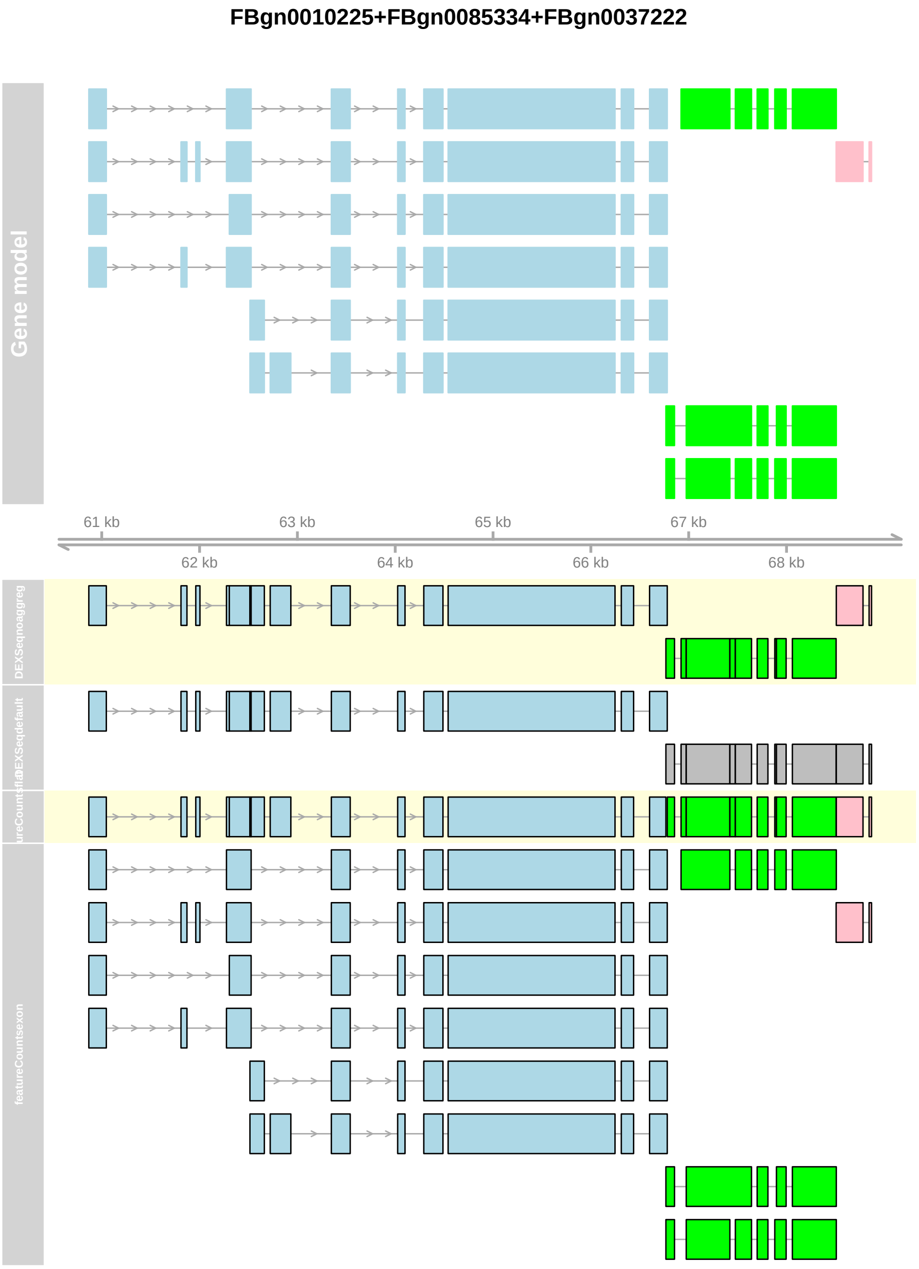

Supplementary Figure 2: Illustration of the differences between bins generated for *DEXSeq-noaggreg*, *DEXSeq-default*, *featureCounts-flat* and *featureCounts-exon*, for three overlapping genes on different strands (blue gene on positive strand, green and pink on negative strand). The top panel shows the gene models for the three genes (transcripts from the different genes are colored in different colors). The panels below the genome coordinate track show the counting bins defined by each method (from top to bottom: *DEXSeq-noaggreg*, *DEXSeq-default*, *featureCounts-flat* and *featureCounts-exon*). Bins assigned to different genes/complexes are colored in different colors. Here, *DEXSeq-default* merges the overlapping genes on the same strand into a complex and keeps the gene on the opposite strand separate. *DEXSeq-noaggreg* excludes the region overlapping both the green and the pink gene (since they are on the same strand). For *featureCounts-flat*, we instead exclude all regions overlapping at least two genes (regardless of strand) and defines bins based only on remaining regions. *featureCounts-exon* uses the original exons as counting bins.

2 Comparison of the two transcriptomes used for the simulations

The two organisms that were used as basis for the simulations in this study were chosen to explore the impact of different transcriptome characteristics. In Supplementary Table 1 and Supplementary Figure 3 we summarise some of the properties of the two transcriptomes. Most strikingly, the fruit fly (*Drosophila melanogaster*) has (on average) considerably fewer isoforms per gene, but the exons and transcripts are longer than for the human transcriptome (*Homo sapiens*). There is a higher degree of overlap among the human genes, manifested by the larger fraction of genes and counting bins that the default DEXSeq pre-processing workflow assigns to gene complexes consisting of more than one gene. There is also a large difference in the fraction of isoforms that were considered non-expressed in the two real data sets used as the basis for the simulations. While only around 13% of the fruit fly transcripts had an estimated TPM of 0 by RSEM, this was the case for almost 60% of the human transcripts.

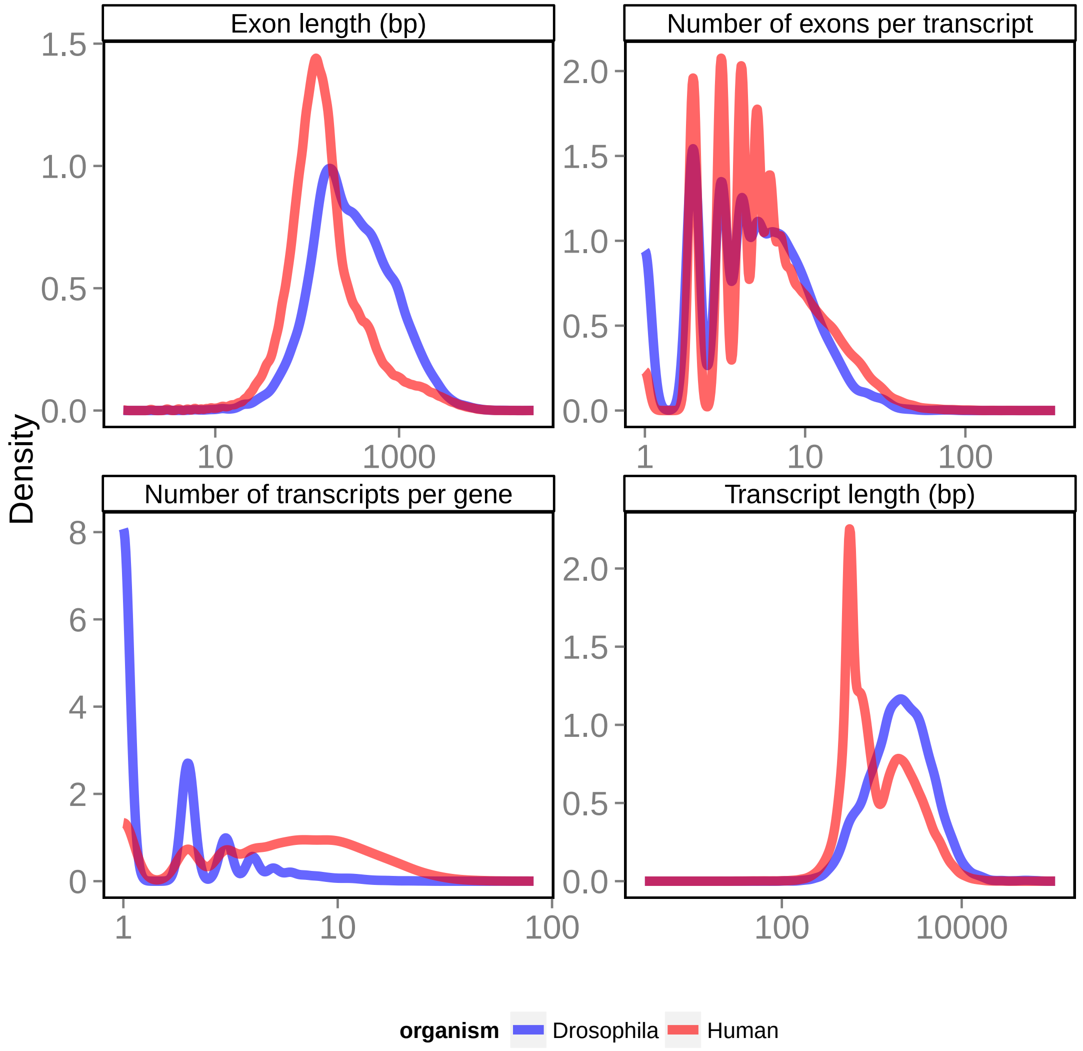

Supplementary Figure 3: Summary of the two transcriptomes used as basis for the simulations (Drosophila (fruit fly) and human). The human transcriptome has more transcripts per gene (lower left), while the number of exons per transcript is similar for the two organisms (upper right). The fruit fly genome has longer exons and transcripts than the human genome (upper left and lower right).

Supplementary Table 1: Summary statistics for the two transcriptomes used as basis for the simulations. The Ensembl gtf files that are used for the simulation contain multiple instances of exons that are part of multiple isoforms. The 'Number of exons' given in the table represents the total number of exons in the respective gtf files (counting each instance of repeated exons), while the 'Number of unique exons' represents the number of unique exon IDs in the file. The 'Number of non-expressed transcripts' and 'Fraction of non-expressed transcripts' are based on the RSEM TPM estimates from the real RNA-seq data sets, which were used as the basis for the simulation.

	D. melanogaster	*H. sapiens*
Number of genes	13937	20410
Number of transcripts	26951	145342
Number of exons	159182	1069500
Number of unique exons	77310	566352
Min number of isoforms per gene	1	1
Median number of isoforms per gene	1	5
Mean number of isoforms per gene	1.93	7.12
Max number of isoforms per gene	74	82
Min number of exons per isoform	1	1
Median number of exons per isoform	4	5
Mean number of exons per isoform	5.91	7.36
Max number of exons per isoform	82	363
Number of genes and complexes (*DEXSeq-default*)	13569	19099
Number of complexes (*DEXSeq-default*)	341	840
Number of *DEXSeq-default* counting bins	75162	478504
Number of *DEXSeq-default* bins falling in gene complexes	3524	44984
Fraction of *DEXSeq-default* bins falling in gene complexes	0.0469	0.0940
Number of genes and complexes (*casper*)	11505	12544
Number of complexes (*casper*)	1587	2022
Number of *casper* counting bins	555346	3523252
Number of *casper* bins falling in gene complexes	149394	1174239
Fraction of *casper* bins falling in gene complexes	0.269	0.333
Number of genes and complexes (*cuffdiff*)	13569	19102
Number of complexes (*cuffdiff*)	266	829
Number of non-expressed transcripts (TPM=0)	3422	86958
Fraction of non-expressed transcripts	0.127	0.598

3 Simulation and study design details

Supplementary Figure 4 shows a schematic illustration of the simulation and analysis parts of the current study. For the simulation part, we first generate an index for RSEM from the genome fasta and gtf file for the organism of interest. Using a pair of fastq files from a real data set, we then estimate RSEM model parameters and transcript expression levels to use as the basis for the simulation. We use a mean/dispersion relationship estimated from real data (see [12]) to generate sample-wise transcript expression estimates, and also introduce differential transcript usage for 1,000 genes, by switching the relative abundances for the two most abundant isoforms between the conditions. Finally, we use RSEM to simulate paired fastq files with a read length of 101 bp for each of the six samples, as well as a table with the true differential transcript usage (DTU) status for each gene in the simulation.

For the analysis part, we start by preparing annotation files and index files necessary for the subsequent steps. We use DEXSeq to generate two flattened gff files (using default parameters as well as setting `--aggregate='no'` to avoid merging overlapping genes into complexes). We also generate a manually flattened gtf file (using functions from Bioconductor [5]) to use for counting with the Rsubread package [8]. The Rsubread package was also used for counting based on the original gtf file (without flattening). Illustrations of the differences in the counting bins defined by these four approaches for two example genes are given in Supplementary Figures 1 and 2.

MISO requires a gff3 annotation file for indexing, and this was generated using a perl script downloaded from `https://github.com/mbomhoff/bio-utils/blob/master/gtf_to_gff.pl`. Finally, *cuffdiff* requires an annotation file containing tss_id and p_id fields, which was generated using the cuffcompare module of the cufflinks toolbox. We generated index files for kallisto and TopHat2, and aligned the simulated reads with TopHat2 to generate bam files. The preprocessed data were submitted to nine counting methods, one event-based method (*rMATS*) and one assembly-based method (*cuffdiff*). All count matrices were used as input to DEXSeq for differential bin usage testing (blue arrows in Supplementary Figure 4).

The genes in our study were simulated to have the same expression level (in TPM = transcript per million units) in the two conditions. We selected 1,000 genes in each simulation to show differential usage between the conditions. These genes were selected randomly among genes with sufficiently high expression (expected count of at least 500) and at least two sufficiently highly expressed isoforms (relative abundance above 10%). For the genes affected by the differential isoform usage, although the number of simulated transcripts is similar between the conditions, the identity of these transcripts are different (they represent different isoforms). Since the isoforms may have different lengths, the expected read count for these genes may differ between the conditions, even if the TPM is similar. By construction, for the differentially used isoforms, the TPM differs between the conditions. These properties are illustrated in Supplementary Figures 5 and 6 for the fruit fly and human simulations, respectively.

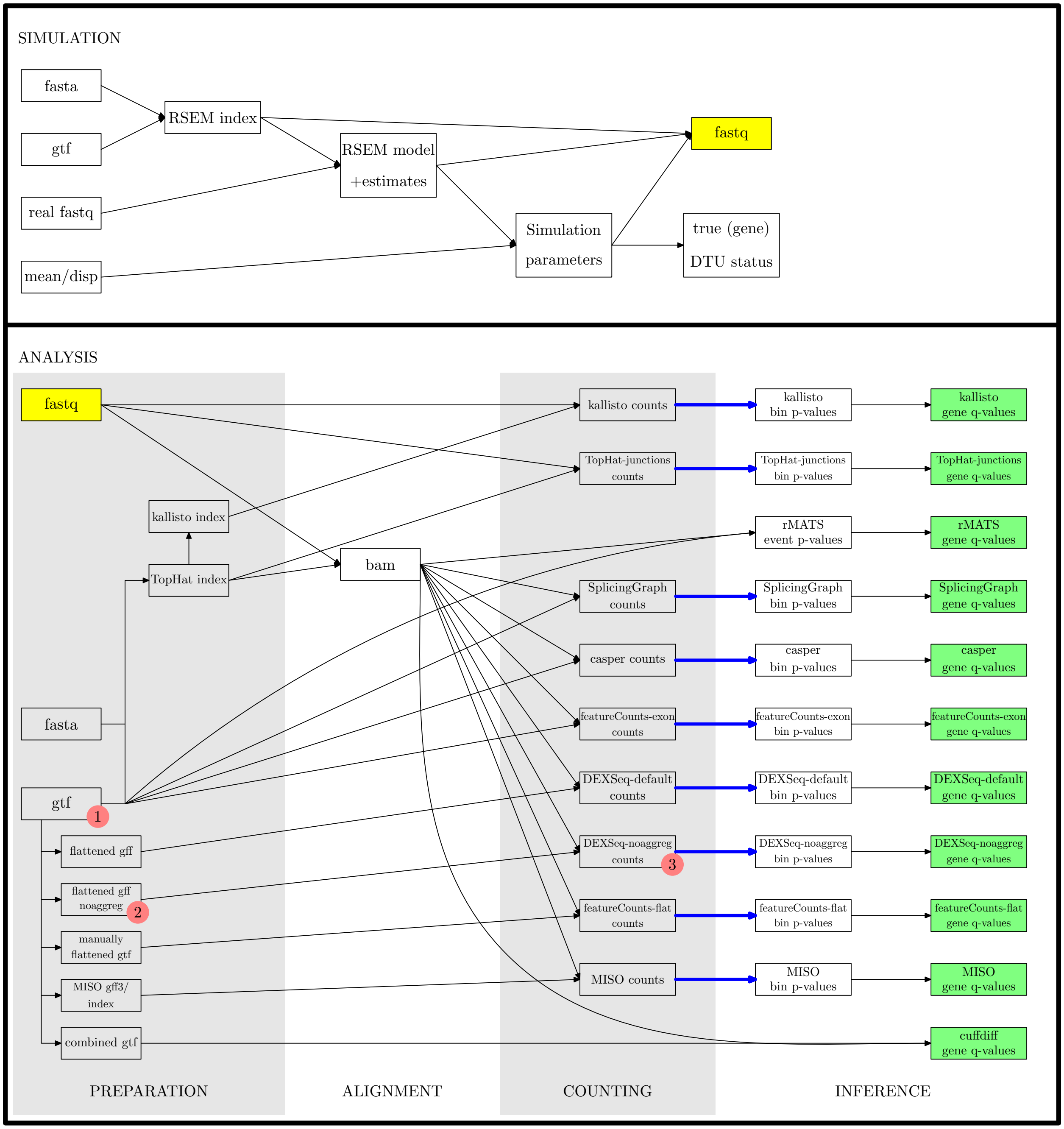

Supplementary Figure 4: Schematic illustration of the simulation and analysis parts of the current study. The yellow boxes represent the simulated fastq files, which were used as input to all DTU detection methods. The blue arrows represent testing for differential bin usage between conditions with DEXSeq, which was done for all nine count matrices. The green boxes contain the final gene-level results, which were compared to the true DTU status obtained from the simulation. The three red circles indicate the modifications that were introduced (independently) to investigate the effects of changes to the annotation catalog. Circle (1) represents the exclusion of 20% of the transcripts to generate an incomplete annotation catalog. All downstream steps (from index building and alignment) were re-run after this modification. Circle (2) represents the isoform-guided filtering performed for *DEXSeq-noaggreg* with the aim of improving the FDR control. Similarly, circle (3) represents the bin-level filtering that was applied to the original *DEXSeq-noaggreg* count matrix and contrasted with the isoform-guided filtering (see Section 14 in this Supplementary Material).

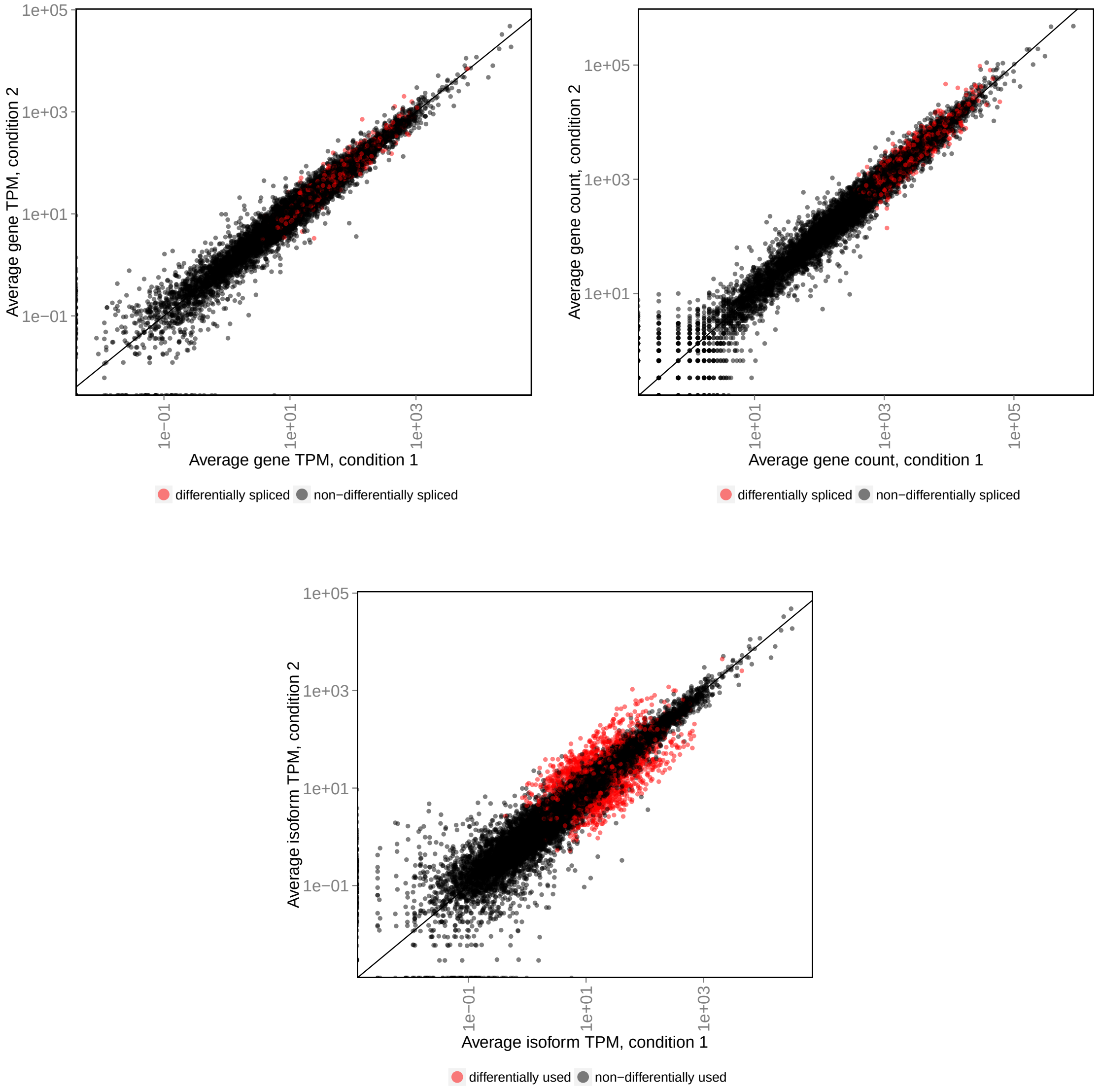

Supplementary Figure 5: Illustration of the average expression levels for genes and transcripts in the two sample conditions, for the fruit fly simulation. The gene-wise TPM (upper left) is similar in the two conditions, for all genes (regardless of differential splicing status). Since the expressed isoforms may have different length, the average gene count (upper right) may potentially be different between the conditions for the differentially spliced genes. However, this effect is weak in the fruit fly simulation shown here. On the transcript-level (lower), the TPM of the differentially used isoforms are clearly different between the two conditions, as expected. The diagonal lines are identity lines.

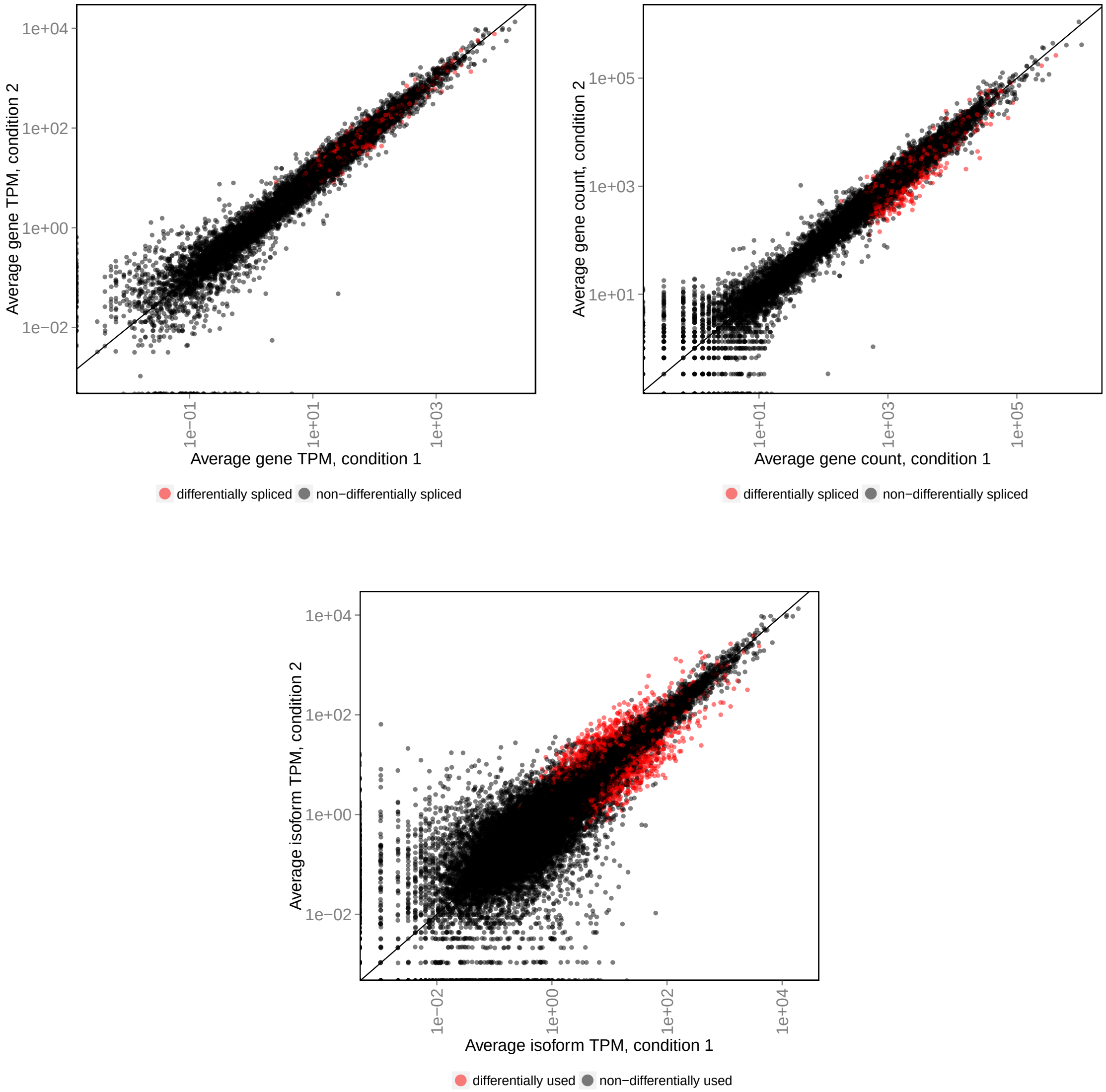

Supplementary Figure 6: Illustration of the average expression levels for genes and transcripts in the two sample conditions, for the human simulation. The gene-wise TPM (upper left) is similar in the two conditions, for all genes (regardless of differential splicing status). Since the expressed isoforms may have different length, the average gene count (upper right) may potentially be different between the conditions for the differentially spliced genes. This effect is visible for some of the genes. On the transcript-level (lower), the TPM of the differentially used isoforms are clearly different between the two conditions, as expected. The diagonal lines are identity lines.

4 Additional information about the counting methods

4.1 MISO

MISO (Mixture of Isoforms, v0.5.3) [6] is a probabilistic method that (in the "isoform-centric" mode) allows to quantify the expression of individual isoforms and (optionally) test for differential usage between samples. Among other information, the MISO output files provides, for each gene, the number of reads that are consistent with any combination of the gene's isoforms (including the number that are not consistent with any isoform). We defined the counting bins as these isoform combinations, excluding the unassignable reads. The MISO quantification is only performed for genes with at least two isoforms, and with at least one assigned read. We did not apply the testing framework provided by MISO, since it is currently not able to accommodate replicates.

4.2 DEXSeq

The python script `dexseq_prepare_annotation.py`, included in the DEXSeq R package (v1.14.0) [1], generates a "flattened" gene model reference file, where the counting bins are defined as disjoint gene parts. In the flattening process, the default behaviour is to merge overlapping genes into complexes (which are named by combining the names of the merged genes). We prepared flattened files using the default settings as well as using the option `--aggregate='no'` where instead of merging overlapping genes, the overlapping parts are excluded and genes are kept separate (see also Supplementary Figures 1 and 2). We used the python script `dexseq_count.py`, also included in the DEXSeq R package, to count the number of reads overlapping each counting bin. A read was allowed to overlap and thereby contribute to the count for multiple counting bins.

4.3 featureCounts

The `featureCounts` function from the Rsubread R package (v1.18.0) works similarly to the native DEXSeq counting, summarising the number of reads falling in given genomic regions [7, 8]. We applied `featureCounts` with two different reference gene model files. First, one that was manually flattened to mimic the flattening performed by DEXSeq, but excluding also regions overlapping between genes on different strands, and second, one which instead considered the original exons as the counting bins, without flattening (see also Supplementary Figures 1 and 2). Reads overlapping multiple exons were assigned to all of them.

4.4 kallisto

Kallisto (v0.42.1) [2] directly quantifies the expression on the transcript level, returning an estimated read count for each isoform. Hence, here the counting bins are defined by the isoforms themselves. To build the transcriptome index needed for the kallisto quantification, we used the transcript fasta file that was created by TopHat in the construction of the transcriptome index for the read alignment.

4.5 SplicingGraphs

Splicing graphs are directed acyclic graphs describing the splicing patterns of genes [4, 10, 13]. Each splicing site (acceptor site, donor site, transcription start site or transcription end site) is represented by a node in the graph (numbered by their position in the transcript) and the edges represent exons and introns connecting the splicing sites. Transcripts are represented as paths in the splicing graph. We used the R package SplicingGraphs (v1.8.1) to generate splicing graphs for the transcripts in our reference files and to assign the reads to their edges (exons or introns), which are used as counting bins. Reads can be assigned to more than one counting bin if they overlap multiple edges. We used the default settings of the SplicingGraphs package, creating splicing graphs and quantifying only genes with at least two isoforms.

4.6 casper

The casper R package (v2.2.0) implements a method for estimating transcript expression that is specifically adapted for paired-end reads [9]. Similarly to DEXSeq, casper first splits the exons for a gene into disjoint exon bins, and merges genes with any overlapping exons. The casper counting bins are, however, not the exon bins themselves but *exon paths*, designed to incorporate more information that is of importance to differential splicing detection. An exon path consists of a pair of exon sequences that are covered by each of the two reads in a pair. By summarising the number of reads following given exon paths rather than individual junctions or exons, casper arguably makes better use of the information provided by reads spanning more than two exons. The combinatorial nature of the path construction implies that the number of counting bins is typically much larger than for the other methods, and consequently the read count for each bin is lower. In the *casper* R package, the exon path counts are used as a basis for estimating transcript expression levels. In this study, however, we examine the usefulness of the intermediate exon path counts as the input to DEXSeq.

4.7 TopHat junction counts

As a byproduct of the TopHat read alignment, the number of reads overlapping each exon junction is calculated. We match these junctions to the reference gene models to assign them to genes, and use the junctions as counting bins. With this counting approach, only the reads overlapping exon junctions are contributing to the count, and thus many reads are not used at all. This may be particularly important if the read length is short compared to the exon length.

5 Comparison of count patterns

To better understand the different behaviours of the counting methods when combined with the DEXSeq inference framework, we compared them in terms of their overall count patterns (Supplementary Figures 7 and 8). In both organisms, *casper* generated considerably more counting bins, and consequently a lower count per bin, than the other methods. The overall number of reads assigned to each gene (the sum of the counts assigned to all its counting bins) by *casper* was similar to many of the other methods (e.g., *DEXSeq-noaggreg* and *featureCounts-flat*). Also *SplicingGraph* and *featureCounts-exon* (in the human simulation) divided the transcriptome into a large number of counting bins, but this was a result of considering also non-coding gene parts (*SplicingGraph*) or keeping many redundant bins (*featureCounts-exon*, see also Supplementary Figures 1 and 2) and did not result in a lower count per bin, but rather a higher gene-level count. The gene-level *TopHat-junctions* counts were lower than for many of the other methods, which was expected since only junction-spanning reads contributed. The difference was more pronounced for the fruit fly simulation, where the exons were long (compared to the reads) and thus relatively few reads spanned junctions.

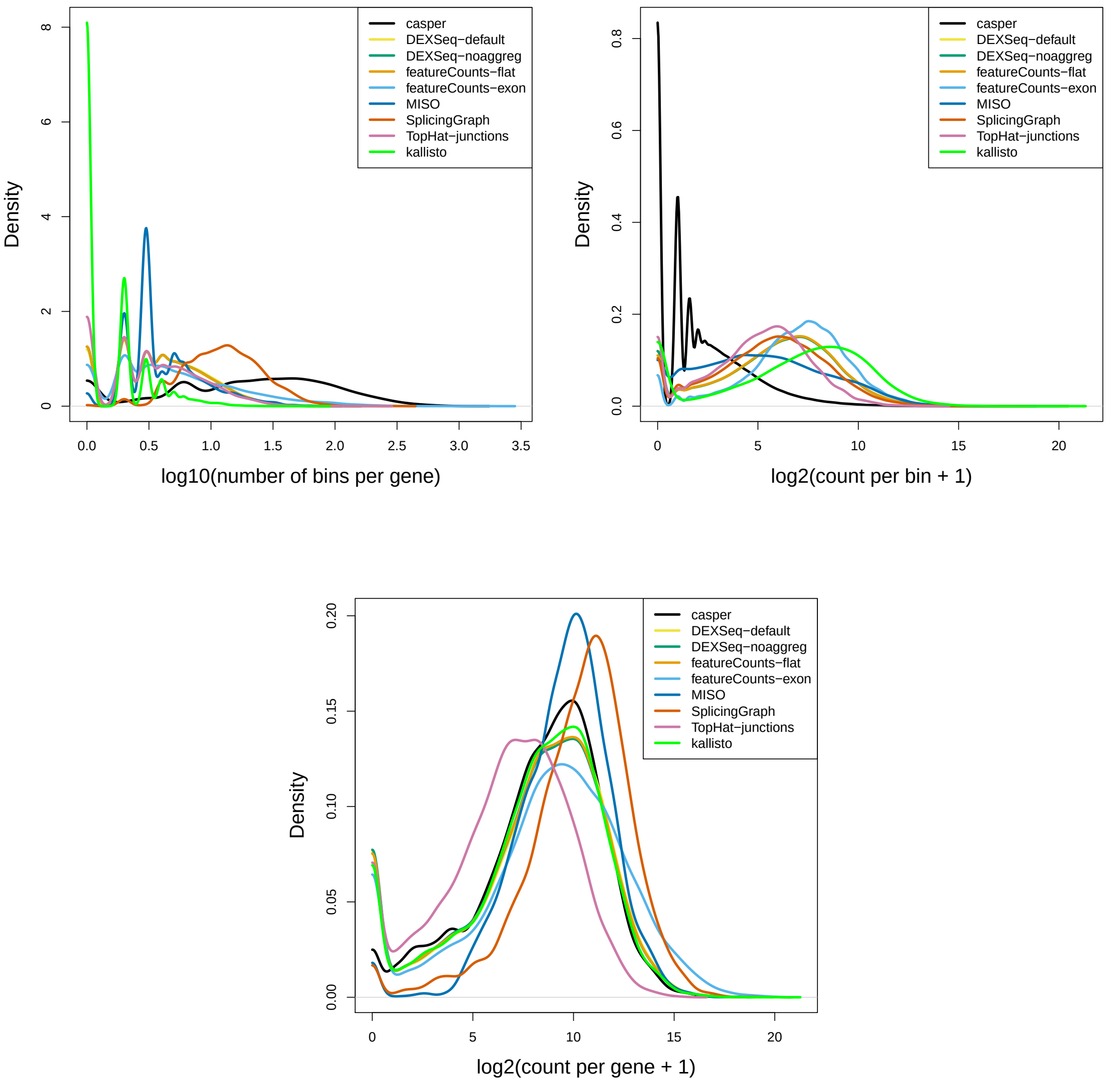

Supplementary Figure 7: Comparison of count patterns among the nine evaluated counting methods, in the fruit fly simulation. The upper left panel shows the distribution of the number of bins per gene, where it is clear that *casper* generates many more bins than most other counting methods, followed by *SplicingGraph*. *kallisto* defines the counting bins as the isoforms themselves, and thus has a high peak at 0 corresponding to genes with a single isoform. The upper right panel shows the distribution of the bin counts for the different methods. The large number of bins for *casper* corresponds to a low count per bin, while the other methods are more similar. The lower panel shows the distribution of the gene-level counts (the sum of the bin counts per gene). As expected, the gene-level counts for *TopHat-junctions* are lower than those for the other methods, since it only considers reads spanning exon junctions.

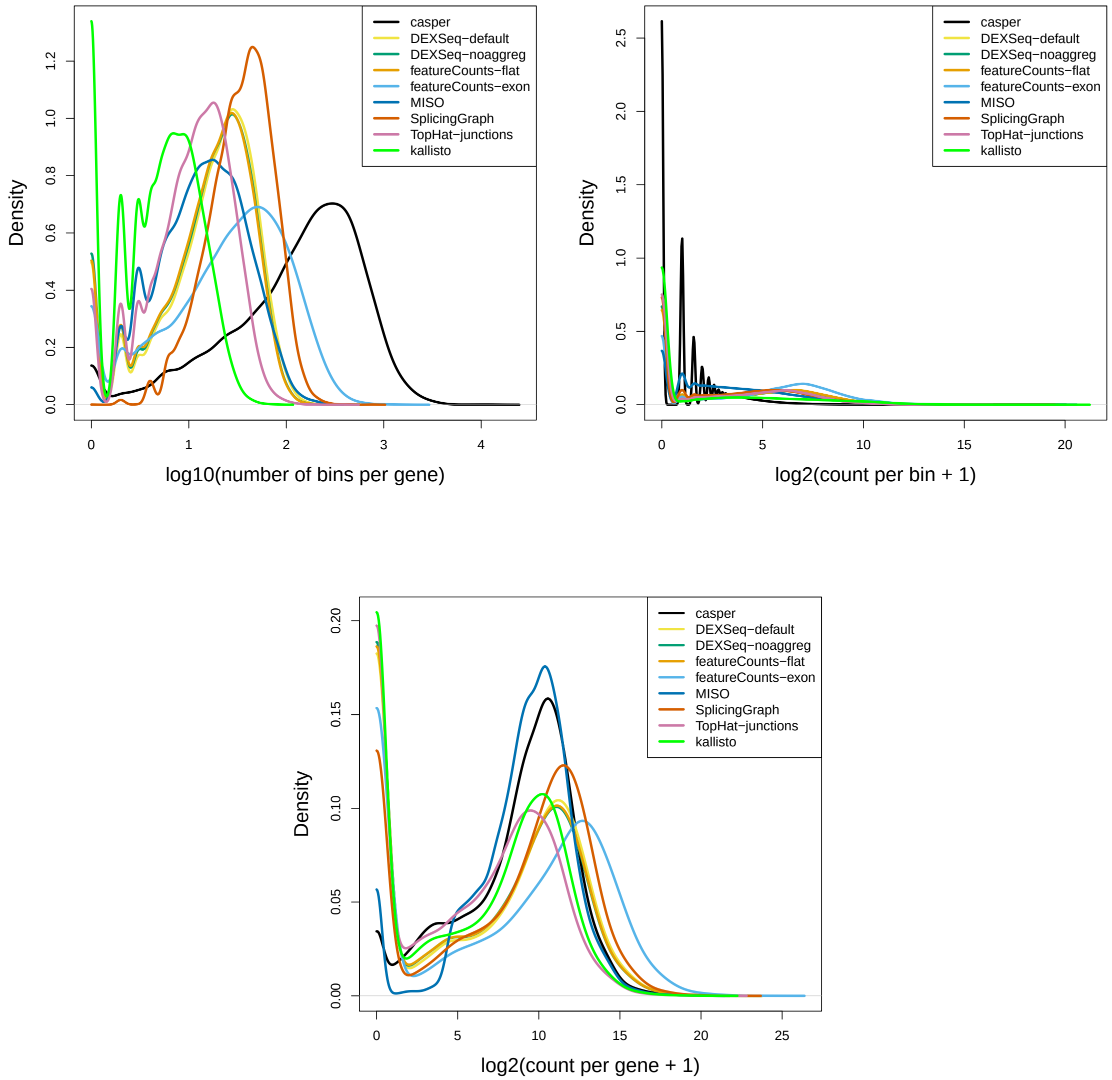

Supplementary Figure 8: Comparison of count patterns among the nine evaluated counting methods, in the human simulation. The upper left panel shows the distribution of the number of bins per gene. As for the fruit fly simulation (Supplementary Figure 7), *casper* generates many more bins than the other counting methods. *kallisto* defines the counting bins as the isoforms themselves, and thus has a peak at 0 corresponding to genes with a single isoform. The upper right panel shows the distribution of the bin counts for the different methods. The large number of bins for *casper* corresponds to a low count per bin, while the other methods are more similar. The lower panel shows the distribution of the gene-level counts (the sum of the bin counts per gene). In this simulation, most reads span exon junctions (the exons are shorter than for the fruit fly simulation), and thus the gene-level counts for *TopHat-junctions* are similar to those from the other methods.

6 Evaluation of cuffdiff with a modified gtf file

As discussed in the main text, *cuffdiff* considers only changes affecting coding parts of genes (that is, differences in the resulting proteins), as opposed to the other methods that consider all differences between exonic regions. It is important to emphasize that also variations in the non-translated parts can have functional implications, since they may, for example, be the binding sites of various proteins. Since a substantial fraction of the introduced differential isoform usages did not impact the coding parts of the genes, we suspected that the difference in perspective would have a considerable effect on the performance estimates for *cuffdiff*, and that this was part of the reason for the apparently low power. Therefore, we also evaluated cuffdiff with an artificial gtf file, where the CDS entries were excluded and replaced by the exons, and similarly the protein IDs were replaced by transcript IDs. As expected, this improved the power of cuffdiff considerably (Supplementary Figures 9-10), while keeping the FDR low. The best performance was seen for genes where the relative abundance difference between the differentially used isoforms were large. For genes where the two differentially used isoforms were expressed at similar levels, the detection power was very low.

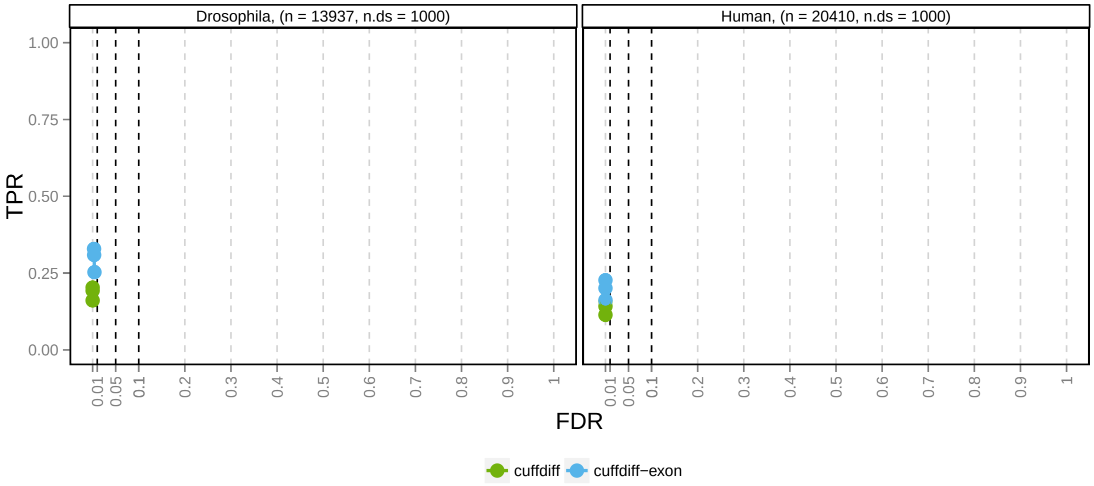

Supplementary Figure 9: Performance evaluation of cuffdiff using the original gtf file (*cuffdiff*), with which we only consider changes in coding sequences, and using a modified, artificial gtf file where CDS entries were replaced by exons and protein IDs with transcript IDs (*cuffdiff-exon*), to force cuffdiff to consider all changes between transcripts. As expected, since many of the introduced changes in differential isoform usage don't lead to changes in the coding sequence, the latter approach showed an increased detection power.

Similarly to *casper* and *DEXSeq-default*, *cuffdiff* also merges overlapping genes into complexes, which also may have a negative influence on the power. Evaluating *cuffdiff* based on only the genes shared with the truth table gave a small improvement, but considerably smaller than for, e.g., casper (for example, the power achieved for the human simulation with a q-value threshold of 0.05 increased from 0.14 to 0.16). The reason for this is that a large part of the improvement seen above for *casper* in particular comes from the exclusion of all genes that are too lowly expressed to be assigned a q-value by DEXSeq (Supplementary Figure 12), and this is not applicable to *cuffdiff*.

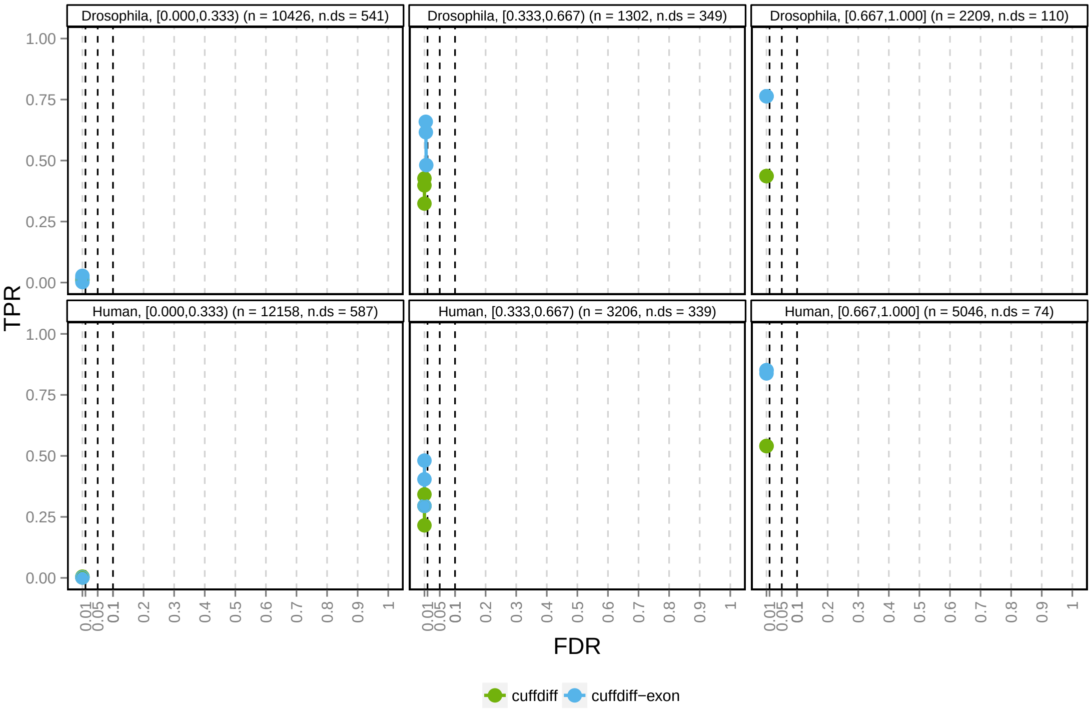

Supplementary Figure 10: Performance evaluation of cuffdiff using the original gtf file (*cuffdiff*), with which we only consider changes in coding sequences, and using a modified, artificial gtf file where CDS entries were replaced by exons and protein IDs with transcript IDs (*cuffdiff-exon*), to force cuffdiff to consider all changes between transcripts. The results are stratified by the difference in relative abundance between the two most abundant isoforms per gene (indicated in the panel headers as, e.g., [0.000,0.333)). As expected, since many of the introduced changes in differential isoform usage don't lead to changes in the coding sequence, the latter approach showed an increased detection power, especially for genes with one dominant isoform (right panel). Changes in relative usage of similarly expressed genes are difficult to detect with cuffdiff (left panels).

7 Performance evaluation stratified by the type of alternative splicing event

Alternative splicing events can be classified into different classes, depending on how the differentially used isoforms differ [11]. Here, we used AStalavista (v3.2) [3] to classify the alternative splicing event for each of the genes affected by differential isoform usage in the two simulations. As for the number of differing basepairs in the previous section, this could only be done for the truly differential genes, and thus we do not report any observed FDR. We report results separately for five major "simple" alternative splicing events (alternative donor sites, alternative acceptor sites, retained intron, skipped exon and mutually exclusive exons), and group all other splicing events (including combinations of the simple ones) together. In the human simulation, only 19 of the 1,000 induced isoform switches corresponded to a simple alternative splicing event, which did not provide enough ground for a reliable analysis, and thus these results are reported only for the fruit fly simulation (Supplementary Figure 11). Again, *featureCounts-exon* showed clear limitations and among the simple alternative splicing events it could only reliably detect skipped exons and mutually exclusive exons. Also *rMATS* could, as expected, only detect simple splicing events, but for these it showed high power. The other methods showed overall similar detection power for all types of alternative splicing events, also complex ones.

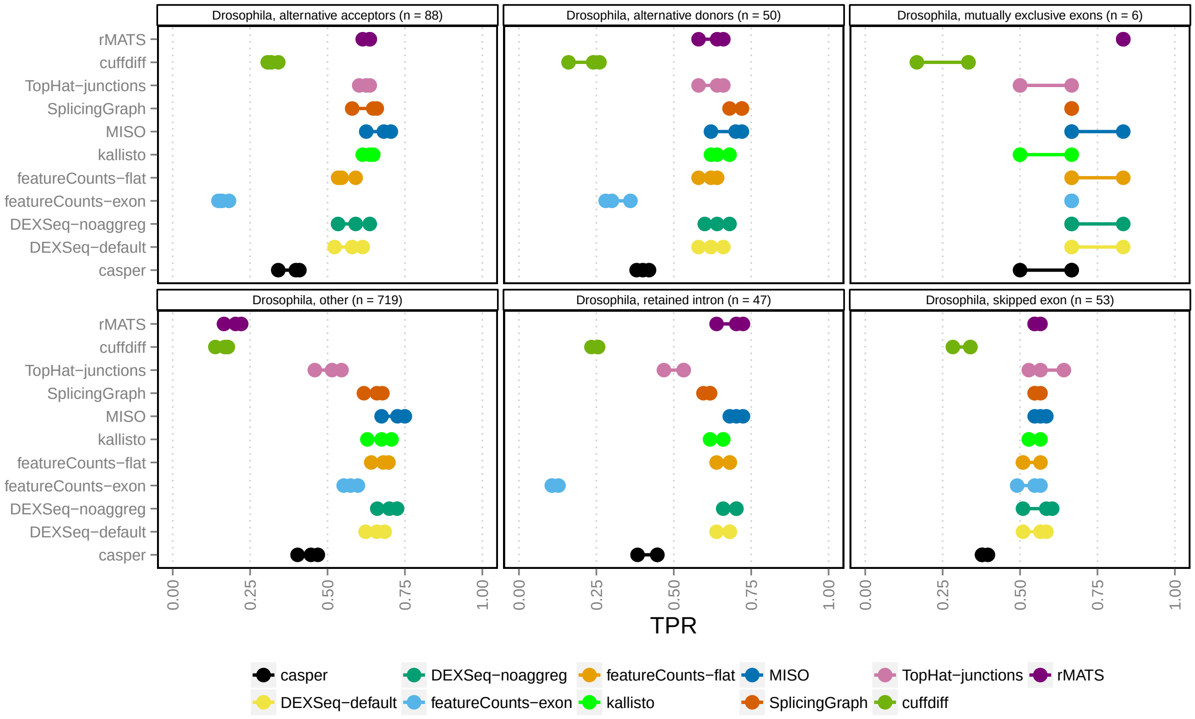

Supplementary Figure 11: Stratification of performance by the type of alternative splicing event defined by the differentially used isoforms. This evaluation is limited to genes affected by differential isoform usage, and to the fruit fly simulation. We extract five types of 'simple' events, and group all other events together. The number of genes in each category is given in the panel headers. The three circles for each method indicate the achieved TPR for three q-value thresholds: 0.01, 0.05 and 0.1. As expected, *rMATS* could only detect the simple events that it is designed to capture. For these event types, however, it had high power. *featureCounts-exon* showed poor detection power for most event types.

8 Performance evaluation based on only genes shared between results and truth

In the main text we evaluated the performance of the methods based on all genes in the annotation catalog. Some methods, more precisely *DEXSeq-default*, *casper* and *cuffdiff*, merge overlapping genes into complexes. Thus, the result table will contain some genes (complexes) that are not present in the annotation catalog and thus can not be categorised as either true positives, true negatives, false positives or false negatives. On the other hand, there are also genes that are present in the annotation catalog but for which we don't have any result. This is partly due to the merging of genes into complexes, and partly (for the counting methods) due to DEXSeq only reporting gene-level q-values for genes where at least one counting bin was expressed to a sufficient level so as to pass the independent filtering step applied by DEXSeq to reliably perform a statistical test. Due to the different characteristics of the counting methods, the latter phenomenon affects a different number of genes for the different methods. Since *rMATS* only considers simple splicing events, only genes harbouring at least one such event will be considered, and no results will be generated for the others. Thus, also here we may be missing a considerable number of genes from the annotation catalog in the result table.

Here, we perform the evaluation of the methods' performances using only the genes that are shared between the annotation catalog and the result table for each method (Supplementary Figure 12). Thus, all genes falling into complexes are excluded, as well as all genes where no counting bin returned a valid p-value or where no test was performed. As expected, the largest improvement in power (compare to Figure 2 in the main manuscript) is seen for *casper*, *DEXSeq-default* and *rMATS*, since all truly differential genes for which no result is generated are excluded, and thus the detected true positives represent a larger fraction of the (retained) truly differential genes.

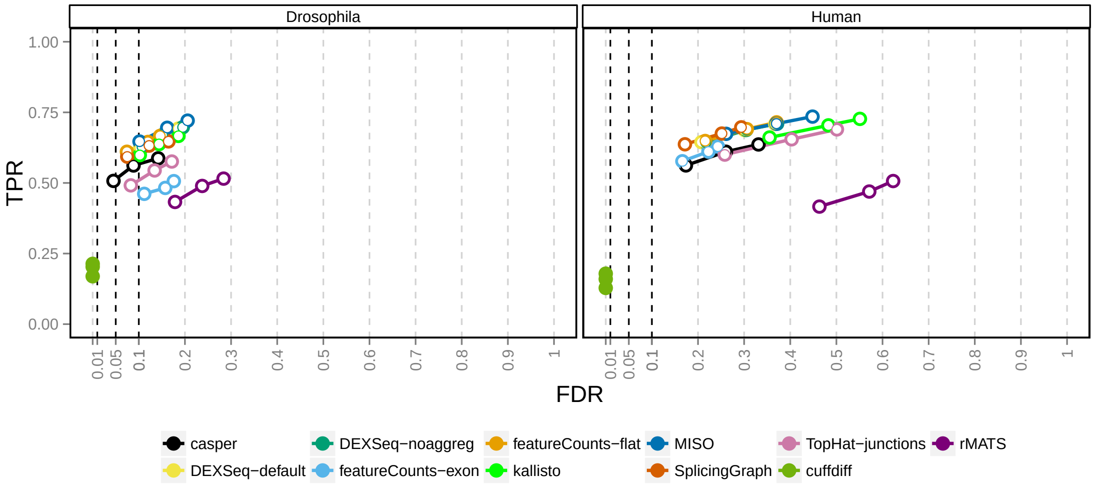

Supplementary Figure 12: Performance evaluation based on only the genes for which the DTU status is known and for which the respective methods return a q-value. The performance of *casper*, *DEXSeq-default* and *rMATS* is improved compared to original evaluation (main text, Figure 2).

9 Performance evaluation stratified by compositional difference between differentially used isoforms

We also investigated the effect of the number of basepairs that distinguished the differentially used isoforms (Supplementary Figure 13). For a given differential gene (truly affected by DTU), we defined the number of differing basepairs between the differentially used isoforms as the total number of basepairs in the union of the two isoforms, minus the total number of basepairs in their intersection. Note that since the number of basepairs differing between the differentially used isoforms was only known for truly differential genes, we could not report the observed FDR in this case. It was clear that *featureCounts-exon* did not manage to satisfactorily capture differential isoform usage for genes where the isoforms differed by less than 100 bp, in any of the simulations. Especially in the human simulation, *kallisto* showed a tendency to be more sensitive than the other methods for detecting switches between genetically similar isoforms. The sensitivity of *rMATS* decreased with increasing difference between the differentially used isoforms, likely since it is limited to detecting simple splicing events.

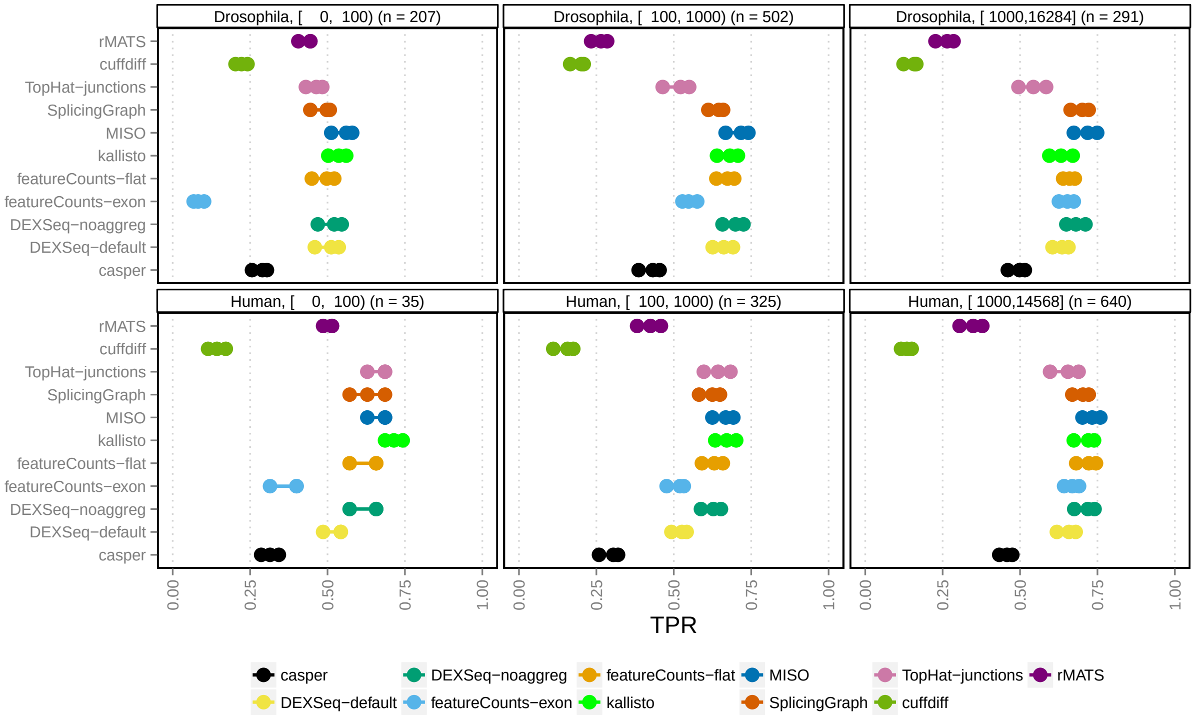

Supplementary Figure 13: Stratification of performance by the compositional difference between the differentially used isoforms. This evaluation is limited to genes that are truly differentially spliced. The compositional difference between two isoforms is defined as the total number of basepairs in the union of the isoforms, minus the total number of basepairs in their intersection. This number is given in the header of each panel, as well as the number of genes in each category. The three circles for each method indicate the achieved TPR for three q-value thresholds: 0.01, 0.05 and 0.1. *featureCounts-exon* was not able to discriminate between very similar isoforms (left panels), while most other methods showed similar power independently of the compositional difference between the differentially used isoforms.

10 rMATS FPs occur due to events with very low prevalence

We observed in the main text that rMATS showed a relatively high false discovery rate, especially for genes with one dominant isoform. To better understand the reason behind this, we extracted the inclusion ratios for all events that were part of truly non-differential genes. Since the genes were non-differential (that is, not affected by differential isoform usage), the average inclusion ratios for the events in each of these genes were similar in the two conditions. For this reason, we represented each event by the average inclusion ratio across all replicates. Comparing the distribution of these average inclusion ratios between the false positives (events with an estimated q-value below 0.05) and true negatives (events with an estimated q-value above 0.05), we see that the false positive events show an enrichment for values close to 0, and a relative depletion of values in between the extremes (Supplementary Figure 14).

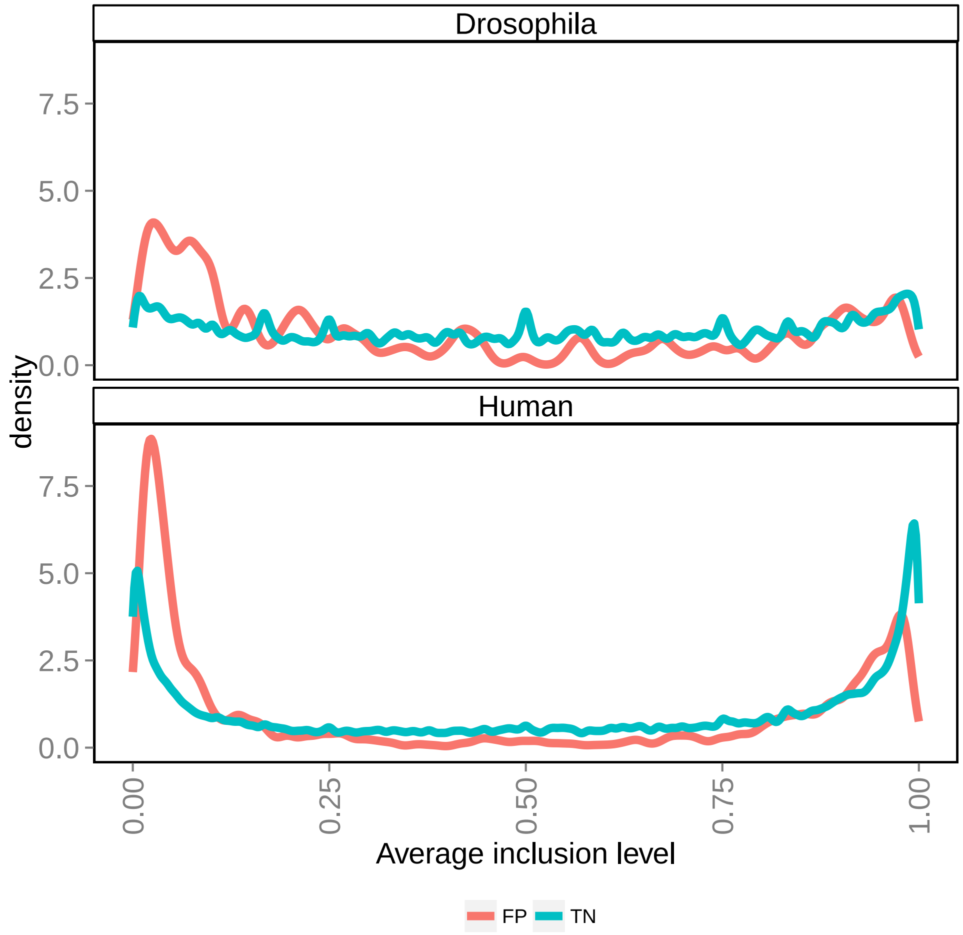

Supplementary Figure 14: Average inclusion level (across the two conditions) of the “false positive events” (events in truly non-differential genes, which are assigned a q-value below 0.05 by *rMATS*) and the “true negative events” (events in truly non-differential genes, which are assigned a q-value above 0.05 by *rMATS*). In both organisms, the false positive events show an enrichment towards the low inclusion levels (close to 0), and a relative depletion of average inclusion levels between 0.25 and 0.75.

11 Performance evaluation stratified by overall gene expression level

The gene expression level (total gene TPM, which as shown in Supplementary Figures 5 and 6 is similar across the two conditions) had a non-negligible effect on the performance of the counting methods, with highly expressed genes showing higher FDR and TPR (Supplementary Figure 15). However, the gene expression level was associated with the number of isoforms for the gene, and thus these effects could not be unambiguously disentangled.

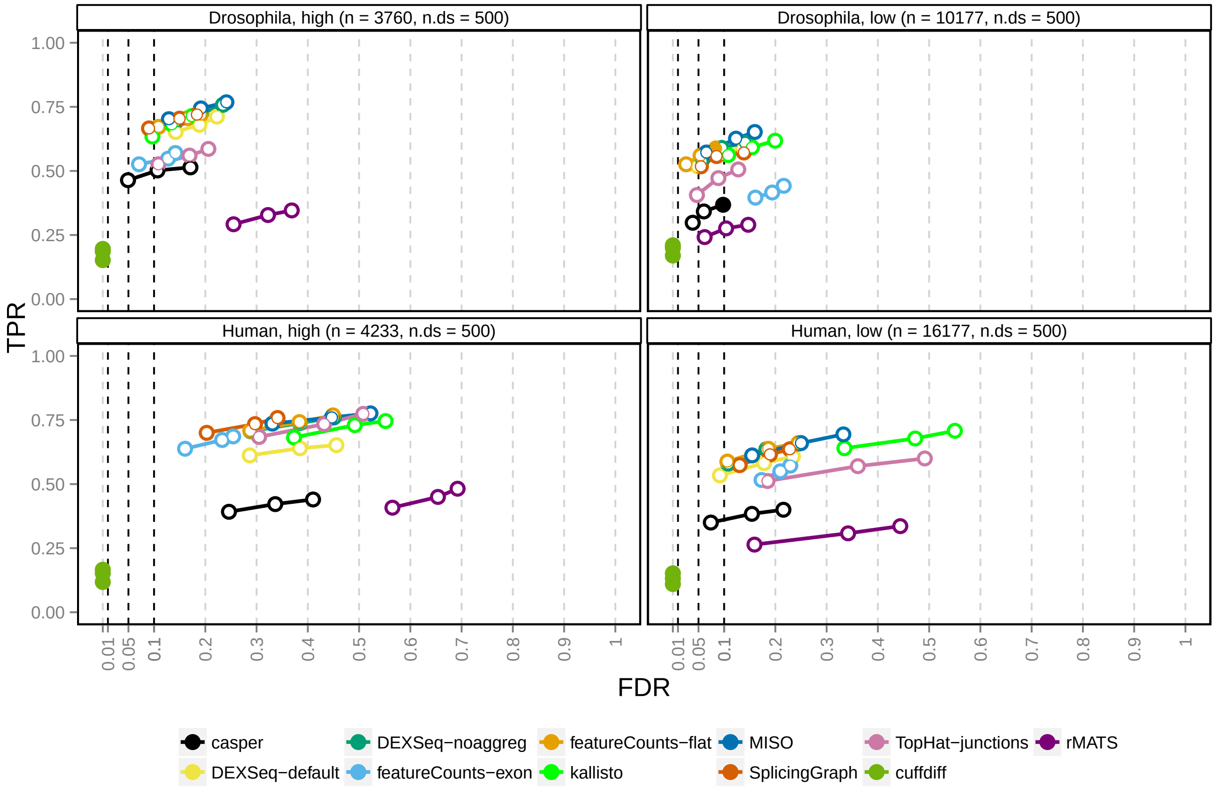

Supplementary Figure 15: Stratification of performance by gene expression level. A 'high' expression level corresponds to TPM exceeding the median of the TPM for the truly differentially spliced genes, and a 'low' expression level correspondingly to TPM below the median TPM for the truly differentially spliced genes. The total number of genes (n) and the number of truly differential genes (n.ds) in each category are given in the panel headers. The three circles for each method indicate the achieved FDR and TPR for three q-value thresholds: 0.01, 0.05 and 0.1. For both organisms, the FDR control is worse and the power somewhat higher for the highly expressed genes.

12 Performance evaluation stratified by incomplete annotation-supplementary figures

In the main text we noted that an incomplete annotation catalog had a more detrimental impact on the performance for the fruit fly simulation than for the human simulation. Here, we expand on this observation and break it down further. First, we note that the main determinant of the performance with the incomplete catalog is the number of isoforms that are retained. In Supplementary Figure 16, the FDR and TPR for the different methods are stratified by the number of retained isoforms per gene (indicated by the number in the header of the panels). The results are also compared to the performance achieved for the same genes with the complete catalog. Note that if no isoforms for a given gene are retained in the catalog, the gene will naturally not be called differential (and thus there are no triangles in the left-most panels). Moreover, the number of truly differential genes among the genes where no isoforms remain, or, for the human simulation, where only one isoform remains, is very low. For the fruit fly simulation, the performance drop among the genes where only one isoform remains in the annotation catalog is large for all counting methods, while no notable performance decrease can be seen for the genes where at least 2 isoforms remain.

In Supplementary Figure 17 we illustrate the effect of the incomplete annotation by the TPR achieved using the complete and the incomplete catalog, stratified by the number of excluded differentially used isoforms. For the genes where no differentially used isoforms is excluded (left panels), no performance decrease is noticed (note that for the truly differential genes, this also means that at least two isoforms are retained). As the number of excluded differentially used isoforms increases (middle and right panels), the performance drops, most notably for the fruit fly simulation. Note, however, that even when both differentially used isoforms are excluded it is sometimes possible to detect the differential transcript usage from the remaining data.

Supplementary Figures 18 and 19 stratify the TPR based on the combination of the number of excluded differentially used isoforms, and the total number of remaining isoforms, for the two organisms. Again, we note that the performance depends mainly on the number of retained isoforms. We also note that the methods that use the transcript structure to define the counting bins (*kallisto*, *SplicingGraph* and *MISO*) are not able to detect the differential transcript usage for genes where only one isoform is retained.

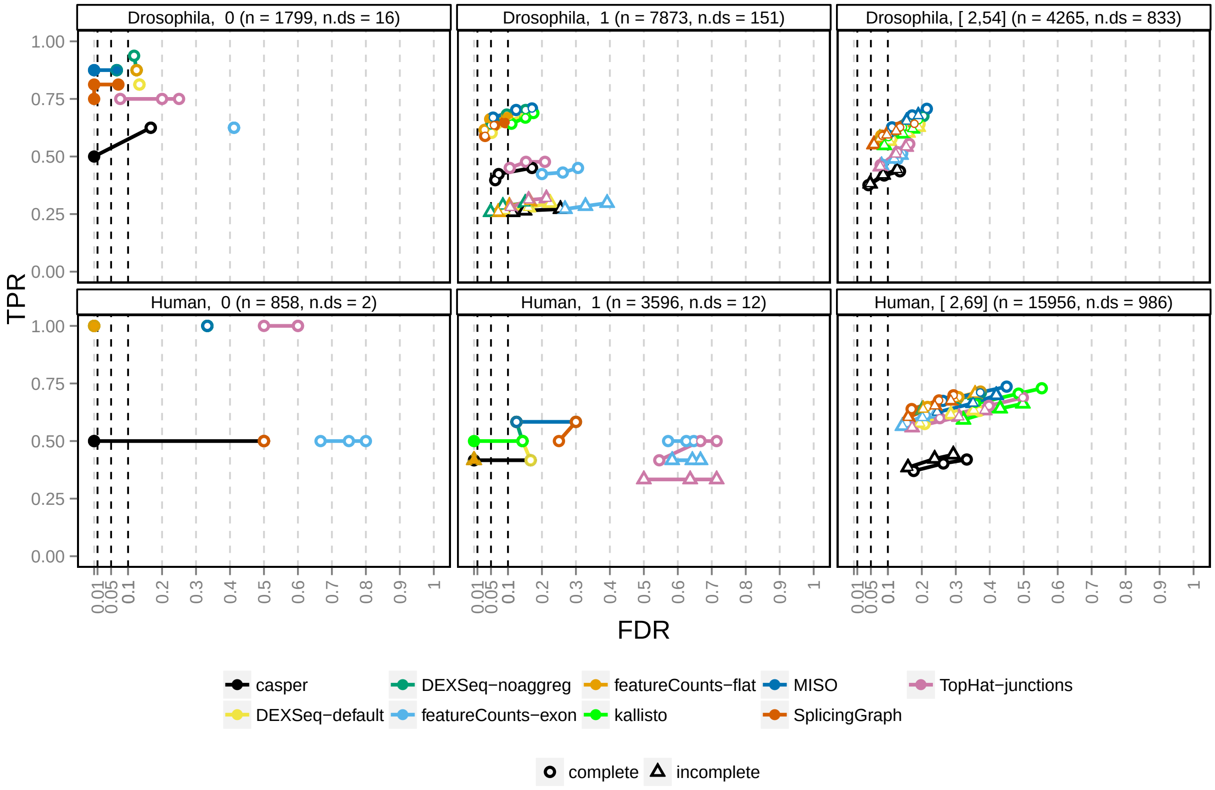

Supplementary Figure 16: The performance of the different counting methods, stratified by the total number of isoforms (per gene) remaining in the annotation catalog after 20% of the transcripts are excluded. The circles correspond to the performance when the full annotation file was used, and the triangles correspond to the performance with the reduced annotation catalog. The number of remaining isoforms per gene, the total number of genes in each stratum (n) and the number of truly differential genes in the stratum (n.ds) are given in the panel headers. The three symbols for each method indicate the achieved FDR and TPR for three q-value thresholds: 0.01, 0.05 and 0.1. The genes where no isoforms remain in the reduced catalog (left panels) are, naturally, not called differential when the reduced catalog is used, and thus there are no triangle symbols in the left panels. We note a big drop in performance for the genes where only one isoform is left in the reduced catalog, while the genes where at least two isoforms remain are not noticeably affected.

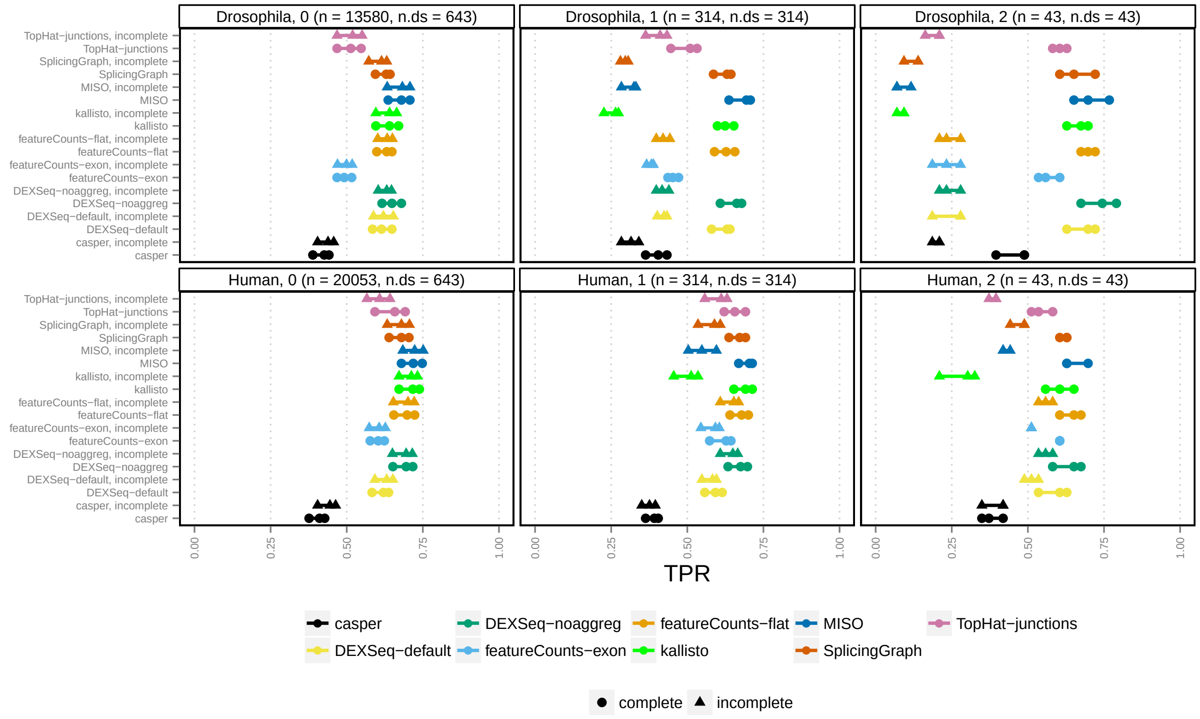

Supplementary Figure 17: The TPR of the different counting methods, stratified by the number of excluded differentially used isoforms (0, 1 or 2; from left to right), when using the complete or incomplete annotation catalog. The number of genes (n) as well as the number of truly differential genes (n.ds) in each category are given in the panel headers. Note that only truly differential genes can have missing differentially used isoforms, and thus n=n.ds for the middle and right panels). The three symbols for each method indicate the achieved TPR for three q-value thresholds: 0.01, 0.05 and 0.1. No reduction in TPR is seen for the genes where no differentially used isoforms are excluded. As the number of excluded differentially used transcripts increases, the performance drops, especially for the fruit fly simulation. However, even for genes where both differentially used isoforms are excluded there is sometimes enough redundancy to detect the differential transcript usage.

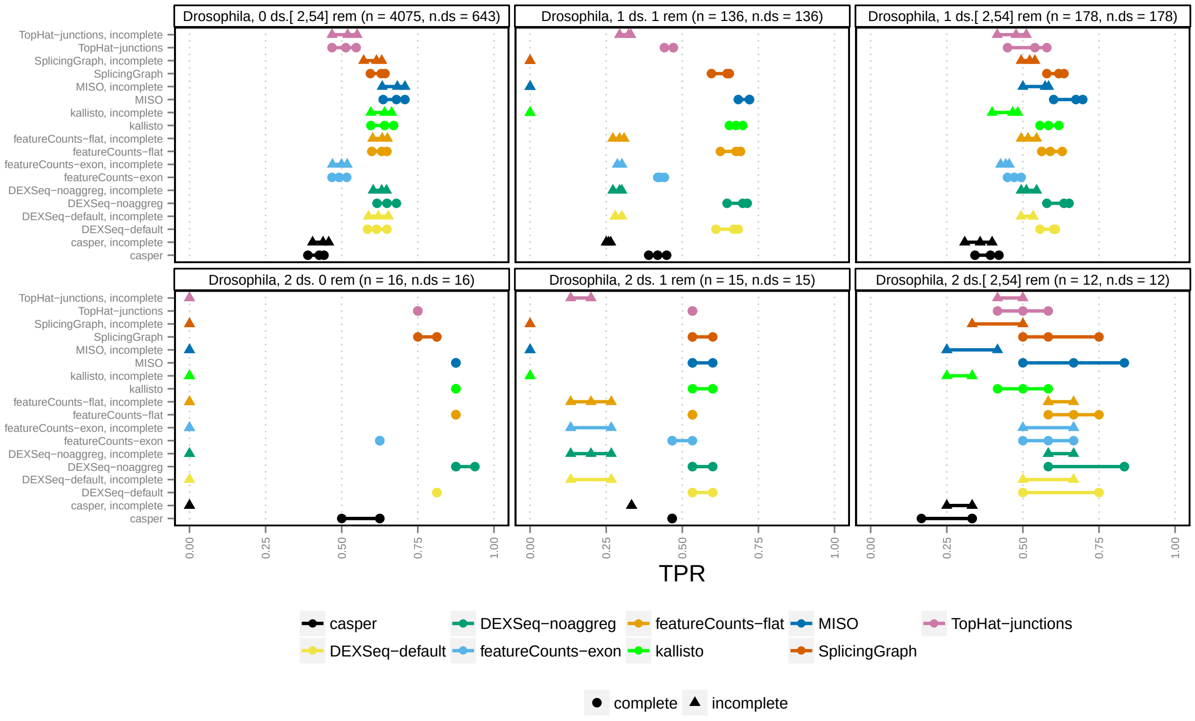

Supplementary Figure 18: The TPR of the different counting methods, stratified by the number of excluded differentially used isoforms ("0 ds", "1 ds" or "2 ds") and the total number of remaining isoforms in the annotation catalog ("0 rem", "1 rem" or "[2,54] rem"), in the fruit fly simulation. The number of genes (n) and the number of truly differential genes (n.ds) in each category are given in the panel headers. The three symbols for each method indicate the achieved TPR for three q-value thresholds: 0.01, 0.05 and 0.1.

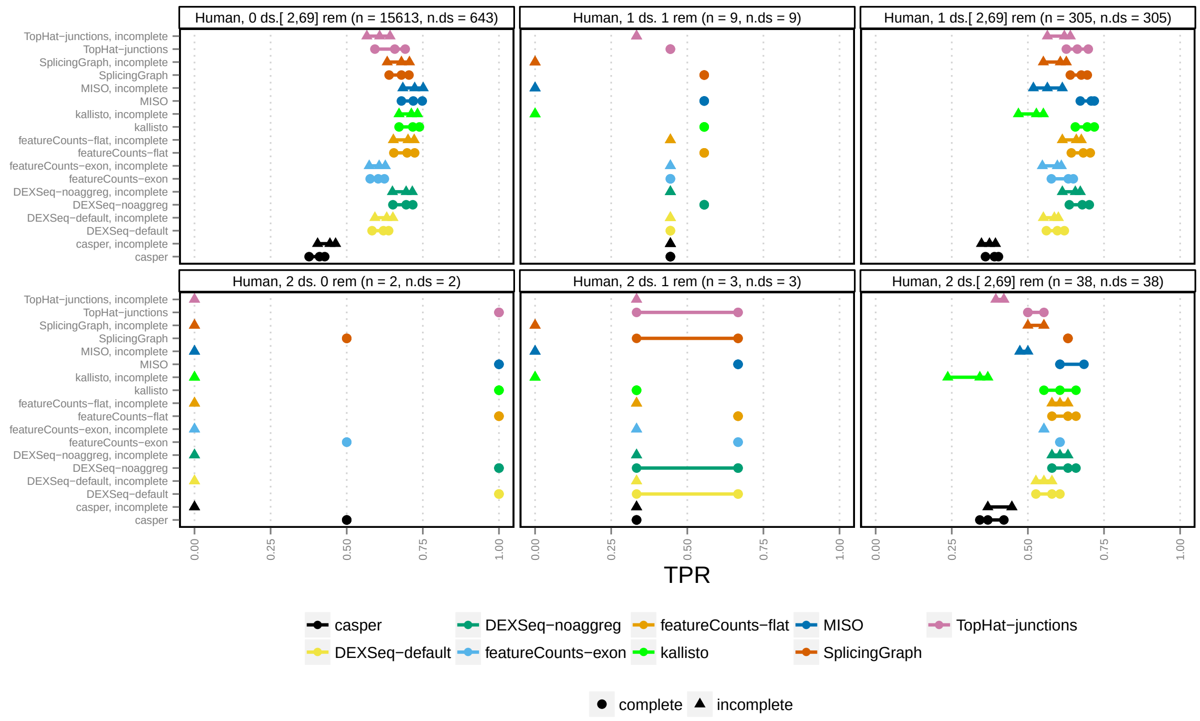

Supplementary Figure 19: The TPR of the different counting methods, stratified by the number of excluded differentially used isoforms ("0 ds", "1 ds" or "2 ds") and the total number of remaining isoforms in the annotation catalog ("0 rem", "1 rem" or "[2,69] rem"), in the human simulation. The number of genes (n) and the number of truly differential genes (n.ds) in each category are given in the panel headers. The three symbols for each method indicate the achieved TPR for three q-value thresholds: 0.01, 0.05 and 0.1.

13 Characterisation of false positives found by *DEXSeq-noaggreg*

In the main manuscript we showed that all counting methods displayed a particularly poor FDR control for genes with one highly dominant isoform (Figure 4), and that the FDR control could be improved by applying a pre-filtering step before the counting (Figure 6). Here, we characterise the false positives found by *DEXSeq-noaggreg* (without the filtering) and investigate the basis for the improved FDR control achieved through filtering.

To characterise the false positives found by *DEXSeq-noaggreg* we calculated the within-gene variance of the counting bin-wise dispersion estimates, and compared to the values obtained for other gene categories (true negatives, true positives and false negatives). The false positives showed a higher dispersion variability than the other categories, especially for the genes with one largely dominant transcript (Supplementary Figure 20) and genes with many counting bins (Supplementary Figure 21).

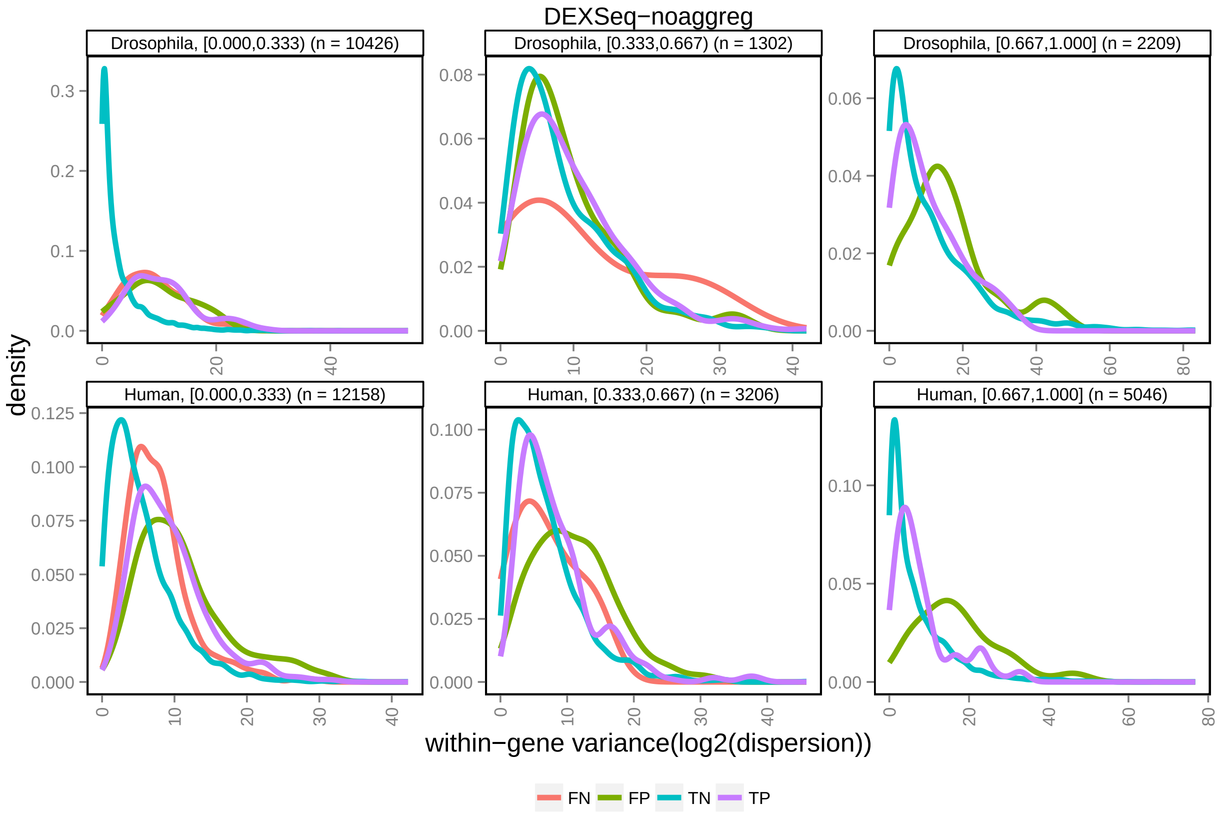

Supplementary Figure 20: Distribution of within-gene variability of counting-bin dispersion estimates obtained by *DEXSeq-noaggreg*, stratified by the relative abundance difference between the two most abundant isoforms and by the gene status (true positive, false positive, true negative and false negative). The false positives show a larger variability in the dispersion estimates than the other gene categories, especially for the genes with one largely dominant isoform (rightmost panels).

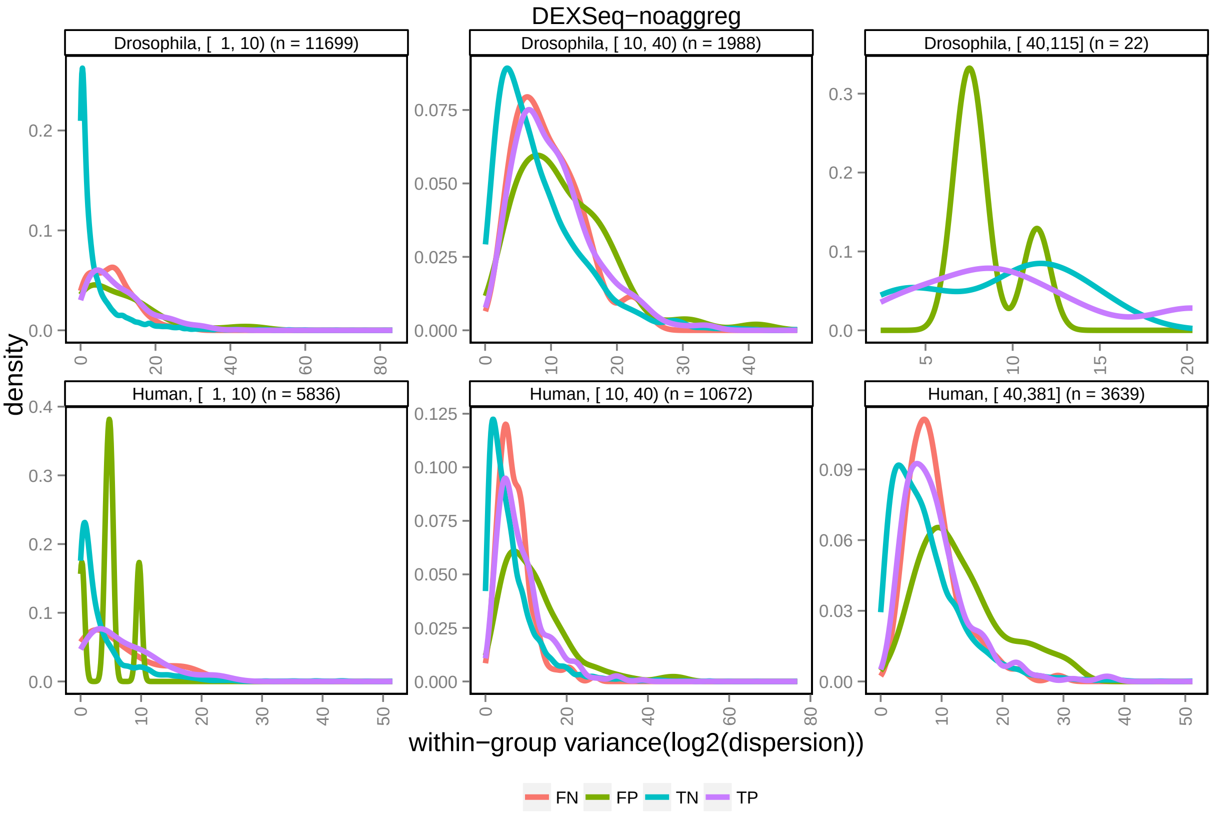

Supplementary Figure 21: Distribution of within-gene variability of counting-bin dispersion estimates obtained by *DEXSeq-noaggreg*, stratified by the number of *DEXSeq-noaggreg* counting bins and by the gene status (true positive, false positive, true negative and false negative). The false positives show a larger variability in the dispersion estimates than the other gene categories, especially for the genes with many counting bins (in the human simulation, lower rightmost panel).

14 Improved FDR control with filtering

14.1 *DEXSeq-noaggreg*

In the main text we showed that by pre-filtering the transcript catalog before constructing the DEXSeq counting bins, excluding all transcripts where the underlying relative abundance was below a certain threshold in both conditions, led to improved control of the FDR. While we could use the underlying relative abundances in our simulated data, these are not available for a real dataset. We therefore suggested that the relative abundances could be estimated by, for example, kallisto [2], and that the resulting estimates could be used to filter the transcript catalog. Here, we evaluate this approach and compare it to other common filtering approaches. These approaches do not exclude features before the counting, but rather exclude counting bins with low or constant expression values, without accounting for the transcript structure. To make the comparison fair, we set the filter thresholds in the bin filtering approaches in such ways that the retained number of bins is the same as for the isoform-based filtering (with abundance estimates by kallisto).

The relative abundance estimates for each isoform were obtained from the sample-wise kallisto abundance estimates. For each sample and each transcript, we calculated the ratio between the TPM for the transcript and the sum of the TPM estimates for all transcripts of the corresponding gene. For each abundance threshold (5%, 10%, 15% and 25%), we excluded all transcripts for which the relative abundance estimate was below the threshold for *all* six samples.

For the bin filtering, we applied six different approaches. All of them take the starting point in the normalised bin counts obtained from DEXSeq (using the `DEXSeq::featureCounts` accessor, with the argument `normalized = TRUE`). Let C_{ik} denote this normalised count in the i'th bin and the k'th sample. We also let G_i denote the collection of bins belonging to the same gene as bin i, and L_i and N_i denote the length of bin i (in kb) and the number of isoforms that contain bin i, respectively. The first filtering approach simply calculates the sum of the normalised counts across all the six samples, and excludes all bins where this sum falls below a given threshold (as mentioned above, the threshold is chosen to give the same number of bins as for the isoform-based filtering). In other words, the filtering statistic for the i'th bin is

$$f_i^1 = \sum_k C_{ik}.$$

A potential disadvantage of this approach is that it doesn't account for the overall expression level of the gene. Thus, we also filtered using the relative bin counts, that is, the normalised bin counts divided by the sum of all the normalised bin counts for the corresponding gene:

$$f_i^2 = \max_k \frac{C_{ik}}{\sum_{j \in G_i} C_{jk}}.$$

The maximum over the samples is introduced to filter out only bins that are lowly expressed across all samples. The f^2 filtering criterion does not, however, account for the different sizes of the bins, and thus may not correspond to the actual relative abundance. Therefore, we also calculated relative bin abundances by first dividing each normalised count with the length of the bin (in kb), and then calculating the ratio between each such abundance measure and the sum of all abundance measures for the gene:

$$f_i^3 = \max_k \frac{\frac{C_{ik}}{L_i}}{\sum_{j \in G_i} \frac{C_{jk}}{L_j}}.$$

When constructing the DEXSeq bin count matrix, a read overlapping multiple bins is allowed to contribute to the read count for each of them. Thus, the count for a bin depends also on the number of isoforms in which it is contained. To account for this, we defined another filter statistic by dividing the normalised counts with the number of isoforms covering the bin instead of its length:

$$f_i^4 = \max_k \frac{\frac{C_{ik}}{N_i}}{\sum_{j \in G_i} \frac{C_{jk}}{N_j}}.$$

We also combine the two corrections (length and number of overlapping isoforms) to create the filter statistic:

$$f_i^5 = \max_k \frac{\frac{C_{ik}}{N_i L_i}}{\sum_{j \in G_i} \frac{C_{jk}}{N_j L_j}}.$$

Finally, we filter using the variance of the (log2-transformed) bin counts across all samples:

$$f_i^6 = var_k(\log_2(C_{ik} + 1)).$$

Supplementary Tables 2 and 3 summarise the filtering thresholds, the number of retained bins and the total count (averaged across all samples) obtained for each filtering criterion. In Supplementary Figures 22-25 we compare the performance achieved with the different filtering criteria. Each figure corresponds to one "filtering threshold", or rather a given number of retained bins. Supplementary Figure 26 shows the performance stratified by the relative abundance difference between the two most abundant isoforms, for the most stringent filtering criteria (corresponding to excluding all isoforms with relative abundances below 25%). As we see in these figures, all filtering methods except the bin variance-based (and all cutoffs) improve the FDR control compared to the unfiltered situation. However, the isoform-guided filtering methods stand out from the bin filtering methods with a lower reduction (or sometimes a slight gain) in power, especially for the human simulation and for the genes where the two most highly expressed isoforms are almost equally expressed (Supplementary Figure 26). The isoform abundances estimated by kallisto perform almost as well as using the underlying abundances from RSEM (which were used for the simulation), which suggests that the isoform-guided filtering is viable also in practice, where the underlying abundances are not known. Among the bin filtering methods, the ones dividing the normalised count by the bin length (f^3 and f^5) perform worst in terms of power loss, likely because they exclude long bins with high read count (Supplementary Tables 2 and 3).

Supplementary Table 2: Summary of the number of retained transcripts and *DEXSeq-noaggreg* counting bins, and the sum of the bin read counts for various filtering methods, in the fruit fly simulation. For the definition of f^1-f^6, see the text.

	Isoform filtering, underlying abundances				
	complete	$\geq 5\%$	$\geq 10\%$	$\geq 15\%$	$\geq 25\%$
# transcripts	26951	20117 (74.6%)	18384 (68.2%)	17323 (64.3%)	15823 (58.7%)
# bins	73981	66554 (90.0%)	64196 (86.8%)	62566 (84.6%)	59649 (80.6%)
tot count	24015599	23777896 (99.0%)	23647396 (98.5%)	23521769 (97.9%)	23029713 (95.9%)

	Isoform filtering, abundance estimates by kallisto				
	complete	$\geq 5\%$	$\geq 10\%$	$\geq 15\%$	$\geq 25\%$
# transcripts	26951	22047 (81.8%)	20163 (74.8%)	18804 (69.8%)	17024 (63.2%)
# bins	73981	68595 (92.7%)	66433 (89.8%)	64605 (87.3%)	61822 (83.6%)
tot count	24015599	23837853 (99.3%)	23754632 (98.9%)	23626187 (98.4%)	23346430 (97.2%)

	Bin filtering, total normalised count				
	complete	$f_i^1 \geq 4.02$	$f_i^1 \geq 11.7$	$f_i^1 \geq 20.6$	$f_i^1 \geq 38.9$
# bins	73981	68594 (92.7%)	66432 (89.8%)	64605 (87.3%)	61822 (83.6%)
tot count	24015599	24014899 (100.0%)	24012076 (100.0%)	24007225 (100.0%)	23993460 (99.9%)

	Bin filtering, relative bin count				
	complete	$f_i^2 \geq 0.24\%$	$f_i^2 \geq 0.66\%$	$f_i^2 \geq 1.14\%$	$f_i^2 \geq 1.98\%$
# bins	73981	68595 (92.7%)	66432 (89.8%)	64604 (87.3%)	61821 (83.6%)
tot count	24015599	24007556 (100.0%)	23967769 (99.8%)	23901931 (99.5%)	23717686 (98.8%)

	Bin filtering, relative bin count accounting for bin size				
	complete	$f_i^3 \geq 0.47\%$	$f_i^3 \geq 1.13\%$	$f_i^3 \geq 1.74\%$	$f_i^3 \geq 2.66\%$
# bins	73981	68594 (92.7%)	66432 (89.8%)	64604 (87.3%)	61822 (83.6%)
tot count	24015599	23950651 (99.7%)	23690182 (98.6%)	23225637 (96.7%)	22358892 (93.1%)

	Bin filtering, relative bin count accounting for number of overlapping isoforms				
	complete	$f_i^4 \geq 0.60\%$	$f_i^4 \geq 1.31\%$	$f_i^4 \geq 1.88\%$	$f_i^4 \geq 2.65\%$
# bins	73981	68594 (92.7%)	66432 (89.8%)	64606 (87.3%)	61821 (83.6%)
tot count	24015599	23998084 (99.9%)	23908166 (99.6%)	23784020 (99.0%)	23477292 (97.8%)

	Bin filtering, relative bin count accounting for bin size and number of overlapping isoforms				
	complete	$f_i^5 \geq 0.92\%$	$f_i^5 \geq 1.52\%$	$f_i^5 \geq 2.02\%$	$f_i^5 \geq 2.81\%$
# bins	73981	68594 (92.7%)	66432 (89.8%)	64605 (87.3%)	61821 (83.6%)
tot count	24015599	23702564 (98.7%)	22958702 (95.6%)	22350166 (93.1%)	21445181 (89.3%)

	Bin filtering, variance of $\log_2$(normalised counts + 1)				
	complete	$f_i^6 \geq 0.029$	$f_i^6 \geq 0.045$	$f_i^6 \geq 0.056$	$f_i^6 \geq 0.072$
# bins	73981	68594 (92.7%)	66432 (89.8%)	64604 (87.3%)	61821 (83.6%)
tot count	24015599	23247326 (96.8%)	22366532 (93.1%)	21596464 (89.9%)	20385864 (84.9%)

Supplementary Table 3: Summary of the number of retained transcripts and *DEXSeq-noaggreg* counting bins, and the sum of the bin read counts for various filtering methods, in the human simulation. For the definition of f^1-f^6, see the text.

	Isoform filtering, underlying abundances				
	complete	$\geq 5\%$	$\geq 10\%$	$\geq 15\%$	$\geq 25\%$
# transcripts	145342	37604 (25.9%)	30151 (20.7%)	25472 (17.5%)	19739 (13.6%)
# bins	468722	211698 (45.2%)	193282 (41.2%)	180439 (38.5%)	160553 (34.3%)
tot count	81568776	65165362 (79.9%)	64069414 (78.5%)	63392031 (77.7%)	61330829 (75.2%)

	Isoform filtering, abundance estimates by kallisto				
	complete	$\geq 5\%$	$\geq 10\%$	$\geq 15\%$	$\geq 25\%$
# transcripts	145342	50334 (34.6%)	40233 (27.7%)	34108 (23.5%)	26151 (18.0%)
# bins	468722	239238 (51.0%)	216265 (46.1%)	201769 (43.0%)	180642 (38.5%)
tot count	81568776	66295556 (81.3%)	64754086 (79.4%)	64025772 (78.5%)	62566691 (76.7%)

	Bin filtering, total normalised count				
	complete	$f_i^1 \geq 39.1$	$f_i^1 \geq 74.7$	$f_i^1 \geq 108.9$	$f_i^1 \geq 174.9$
# bins	468722	239238 (51.0%)	216265 (46.1%)	201769 (43.0%)	180642 (38.5%)
tot count	81568776	81383615 (99.8%)	81169055 (99.5%)	80944942 (99.2%)	80442081 (98.6%)

	Bin filtering, relative abundance				
	complete	$f_i^2 \geq 1.51\%$	$f_i^2 \geq 2.05\%$	$f_i^2 \geq 2.36\%$	$f_i^2 \geq 2.81\%$
# bins	468722	239237 (51.0%)	216265 (46.1%)	201768 (43.0%)	180641 (38.5%)
tot count	81568776	75136307 (92.1%)	68122647 (83.5%)	64035507 (78.5%)	58920931 (72.2%)

	Bin filtering, relative abundance accounting for bin size				
	complete	$f_i^3 \geq 0.90\%$	$f_i^3 \geq 1.24\%$	$f_i^3 \geq 1.48\%$	$f_i^3 \geq 1.88\%$
# bins	468722	239237 (51.0%)	216264 (46.1%)	201768 (43.0%)	180642 (38.5%)
tot count	81568776	62197495 (76.3%)	53762398 (65.9%)	48978079 (60.0%)	42117414 (51.6%)

	Bin filtering, relative abundance accounting for number of overlapping isoforms				
	complete	$f_i^4 \geq 1.46\%$	$f_i^4 \geq 1.83\%$	$f_i^4 \geq 2.06\%$	$f_i^4 \geq 2.43\%$
# bins	468722	239237 (51.0%)	216265 (46.1%)	201769 (43.0%)	180642 (38.5%)
tot count	81568776	71599224 (87.8%)	65660296 (80.5%)	62233309 (76.3%)	56925572 (69.8%)

	Bin filtering, relative abundance accounting for bin size and number of overlapping isoforms				
	complete	$f_i^5 \geq 1.03\%$	$f_i^5 \geq 1.36\%$	$f_i^5 \geq 1.59\%$	$f_i^5 \geq 1.99\%$
# bins	468722	239237 (51.0%)	216265 (46.1%)	201769 (43.0%)	180642 (38.5%)
tot count	81568776	57669094 (70.7%)	49848576 (61.1%)	45460296 (55.7%)	39462172 (48.4%)

	Bin filtering, variance of $\log_2$(normalised counts + 1)				
	complete	$f_i^6 \geq 0.15$	$f_i^6 \geq 0.18$	$f_i^6 \geq 0.21$	$f_i^6 \geq 0.25$
# bins	468722	239237 (51.0%)	216265 (46.1%)	201768 (43.0%)	180872 (38.6%)
tot count	81568776	56154992 (68.8%)	51755644 (63.5%)	48355788 (59.3%)	43636424 (53.5%)

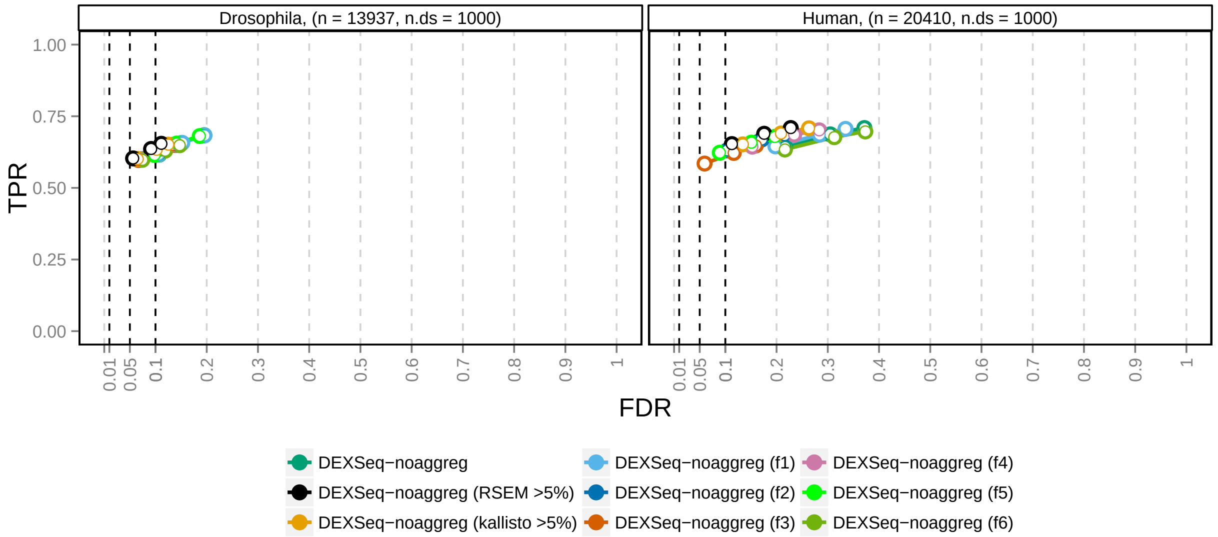

Supplementary Figure 22: Illustration of the performance of *DEXSeq-noaggreg* after different types of (isoform-guided and bin-based) filtering. *DEXSeq-noaggreg* corresponds to the unfiltered analysis. The *DEXSeq-noaggreg (RSEM >5%)* corresponds to filtering out all isoforms with relative abundance below 5% in both conditions in the RSEM estimates from real data, underlying the simulations. *DEXSeq-noaggreg (kallisto >5%)* are the results obtained after excluding all isoform with estimated relative abundance below 5% in all six samples according to kallisto. Both these filterings were performed before the DEXSeq counting bin definition and read counting. The remaining filtering approaches (f1-f6) are applied on the original *DEXSeq-noaggreg* bin counts, using the filter criteria f^1-f^6 defined in the text. The filter thresholds for these methods were defined in such a way that the total number of bins were the same as for *DEXSeq-noaggreg (kallisto >5%)* (see Supplementary Tables 2 and 3). All filtering methods except f^6 improve the FDR control compared to the unfiltered analysis, but the isoform-guided filtering approaches provide the smallest reduction in power. Moreover, the estimated relative abundances from kallisto give almost as good results as using the underlying relative abundances used for the simulation.

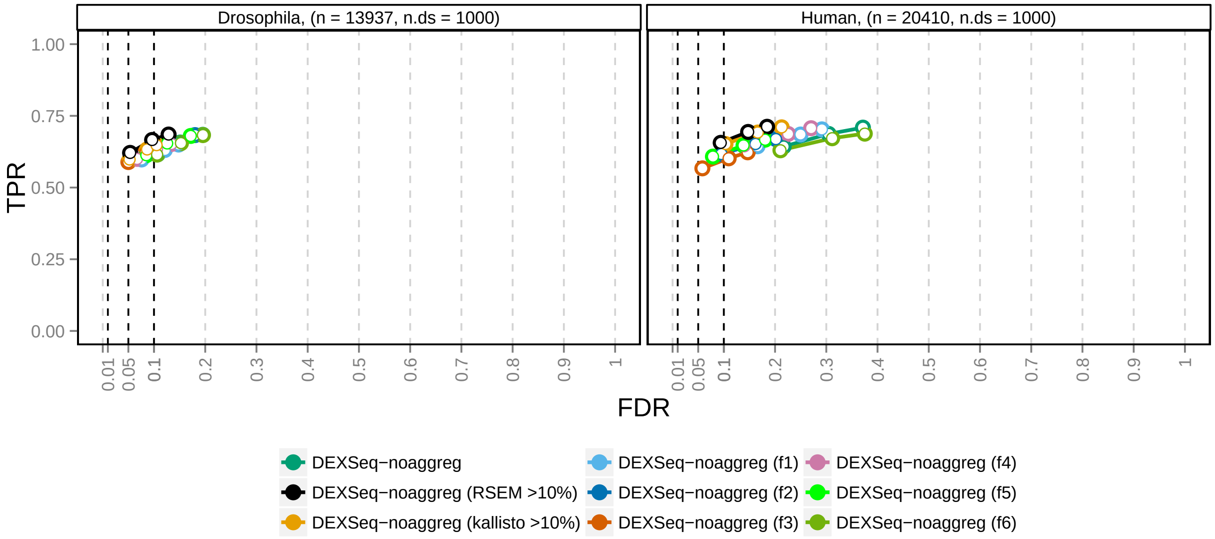

Supplementary Figure 23: Illustration of the performance of *DEXSeq-noaggreg* after different types of (isoform-guided and bin-based) filtering. *DEXSeq-noaggreg* corresponds to the unfiltered analysis. The *DEXSeq-noaggreg (RSEM >10%)* corresponds to filtering out all isoforms with relative abundance below 10% in both conditions in the RSEM estimates from real data, underlying the simulations. *DEXSeq-noaggreg (kallisto >10%)* are the results obtained after excluding all isoform with estimated relative abundance below 10% in all six samples according to kallisto. Both these filterings were performed before the DEXSeq counting bin definition and read counting. The remaining filtering approaches (f1-f6) are applied on the original *DEXSeq-noaggreg* bin counts, using the filter criteria f^1-f^6 defined in the text. The filter thresholds for these methods were defined in such a way that the total number of bins were the same as for *DEXSeq-noaggreg (kallisto >10%)* (see Supplementary Tables 2 and 3). All filtering methods except f^6 improve the FDR control compared to the unfiltered analysis, but the isoform-guided filtering approaches provide the smallest reduction in power. Moreover, the estimated relative abundances from kallisto give almost as good results as using the underlying relative abundances used for the simulation.

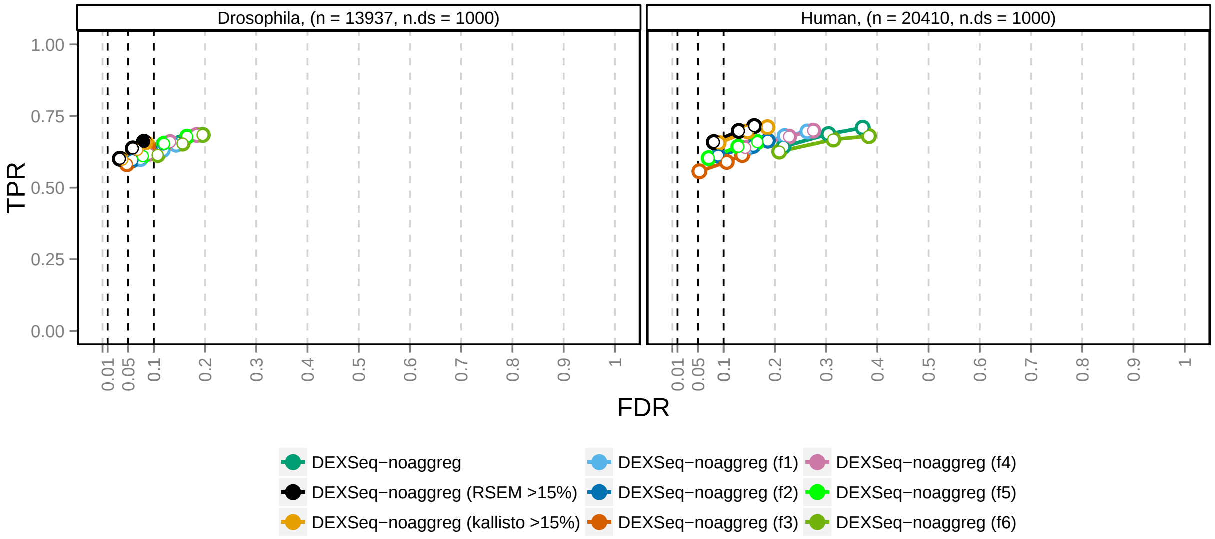

Supplementary Figure 24: Illustration of the performance of *DEXSeq-noaggreg* after different types of (isoform-guided and bin-based) filtering. *DEXSeq-noaggreg* corresponds to the unfiltered analysis. The *DEXSeq-noaggreg (RSEM >15%)* corresponds to filtering out all isoforms with relative abundance below 15% in both conditions in the RSEM estimates from real data, underlying the simulations. *DEXSeq-noaggreg (kallisto >15%)* are the results obtained after excluding all isoform with estimated relative abundance below 15% in all six samples according to kallisto. Both these filterings were performed before the DEXSeq counting bin definition and read counting. The remaining filtering approaches (f1-f6) are applied on the original *DEXSeq-noaggreg* bin counts, using the filter criteria f^1-f^6 defined in the text. The filter thresholds for these methods were defined in such a way that the total number of bins were the same as for *DEXSeq-noaggreg (kallisto >15%)* (see Supplementary Tables 2 and 3). All filtering methods except f^6 improve the FDR control compared to the unfiltered analysis, but the isoform-guided filtering approaches provide the smallest reduction in power. Moreover, the estimated relative abundances from kallisto give almost as good results as using the underlying relative abundances used for the simulation.

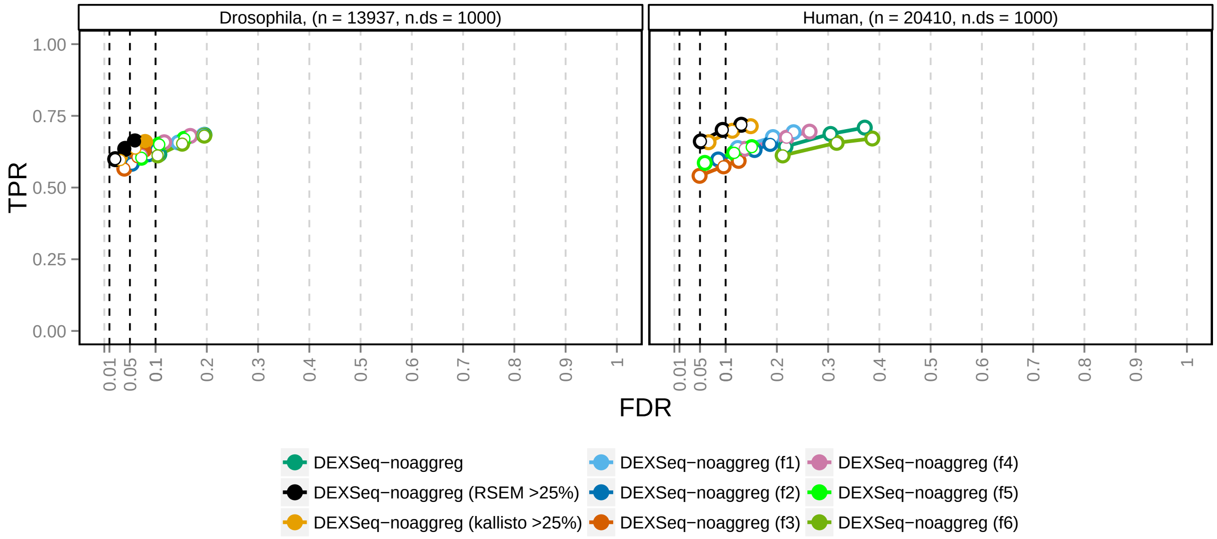

Supplementary Figure 25: Illustration of the performance of *DEXSeq-noaggreg* after different types of (isoform-guided and bin-based) filtering. *DEXSeq-noaggreg* corresponds to the unfiltered analysis. The *DEXSeq-noaggreg (RSEM >25%)* corresponds to filtering out all isoforms with relative abundance below 25% in both conditions in the RSEM estimates from real data, underlying the simulations. *DEXSeq-noaggreg (kallisto >25%)* are the results obtained after excluding all isoform with estimated relative abundance below 25% in all six samples according to kallisto. Both these filterings were performed before the DEXSeq counting bin definition and read counting. The remaining filtering approaches (f1-f6) are applied on the original *DEXSeq-noaggreg* bin counts, using the filter criteria f^1-f^6 defined in the text. The filter thresholds for these methods were defined in such a way that the total number of bins were the same as for *DEXSeq-noaggreg (kallisto >25%)* (see Supplementary Tables 2 and 3). All filtering methods except f^6 improve the FDR control compared to the unfiltered analysis, but the isoform-guided filtering approaches provide the smallest reduction in power. Moreover, the estimated relative abundances from kallisto give almost as good results as using the underlying relative abundances used for the simulation.

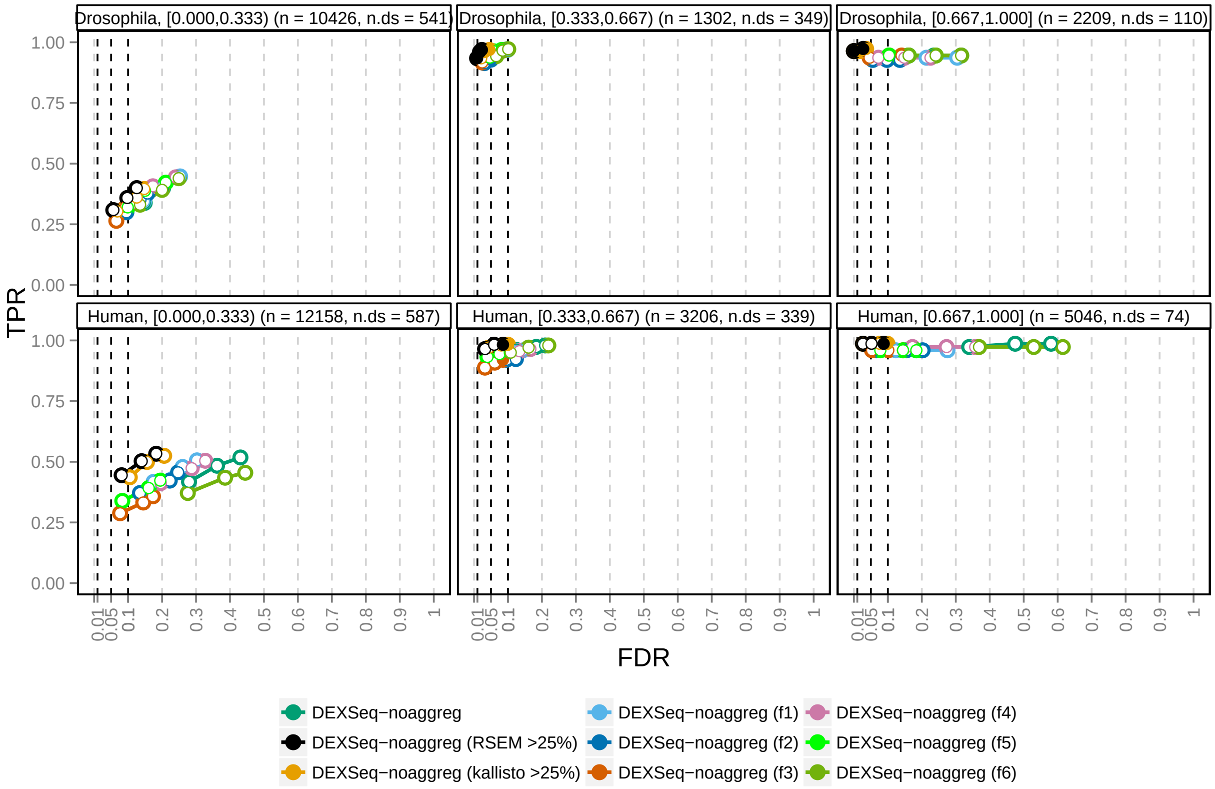

Supplementary Figure 26: Illustration of the performance of *DEXSeq-noaggreg* after different types of (isoform-guided and bin-based) filtering, stratified by the relative abundance difference between the two most highly abundant isoforms. *DEXSeq-noaggreg* corresponds to the unfiltered analysis. The *DEXSeq-noaggreg (RSEM >25%)* corresponds to filtering out all isoforms with relative abundance below 25% in both conditions in the RSEM estimates from real data, underlying the simulations. *DEXSeq-noaggreg (kallisto >25%)* are the results obtained after excluding all isoform with estimated relative abundance below 25% in all six samples according to kallisto. Both these filterings were performed before the DEXSeq counting bin definition and read counting. The remaining filtering approaches (f1-f6) are applied on the original *DEXSeq-noaggreg* bin counts, using the filter criteria f^1-f^6 defined in the text. The filter thresholds for these methods were defined in such a way that the total number of bins were the same as for *DEXSeq-noaggreg (kallisto >25%)* (see Supplementary Tables 2 and 3). All filtering methods except f^6 improve the FDR control compared to the unfiltered analysis, but the isoform-guided filtering approaches provide the smallest reduction in power, especially for the genes where the two most highly abundant isoforms are expressed at similar levels (left panels). Moreover, the estimated relative abundances from kallisto give almost as good results as using the underlying relative abundances used for the simulation.

14.1.1 FPs differing between original and 5% filtered with DEXSeq-noaggreg

In the main text we proposed a simple isoform pre-filtering approach to address the high false discovery rates seen, mainly, in the human simulation with the counting methods. Already excluding all isoforms with relative abundance below 5% in both conditions before constructing the DEXSEq counting bins improved the FDR considerably, without loss of power (see Figure 6). By inspecting the false positives found uniquely when using the original annotation file, or uniquely when using the filtered annotation file, we can increase our understanding of the reasons for the improvement. Here we show a few examples from each of these categories (Supplementary Figures 27-32).

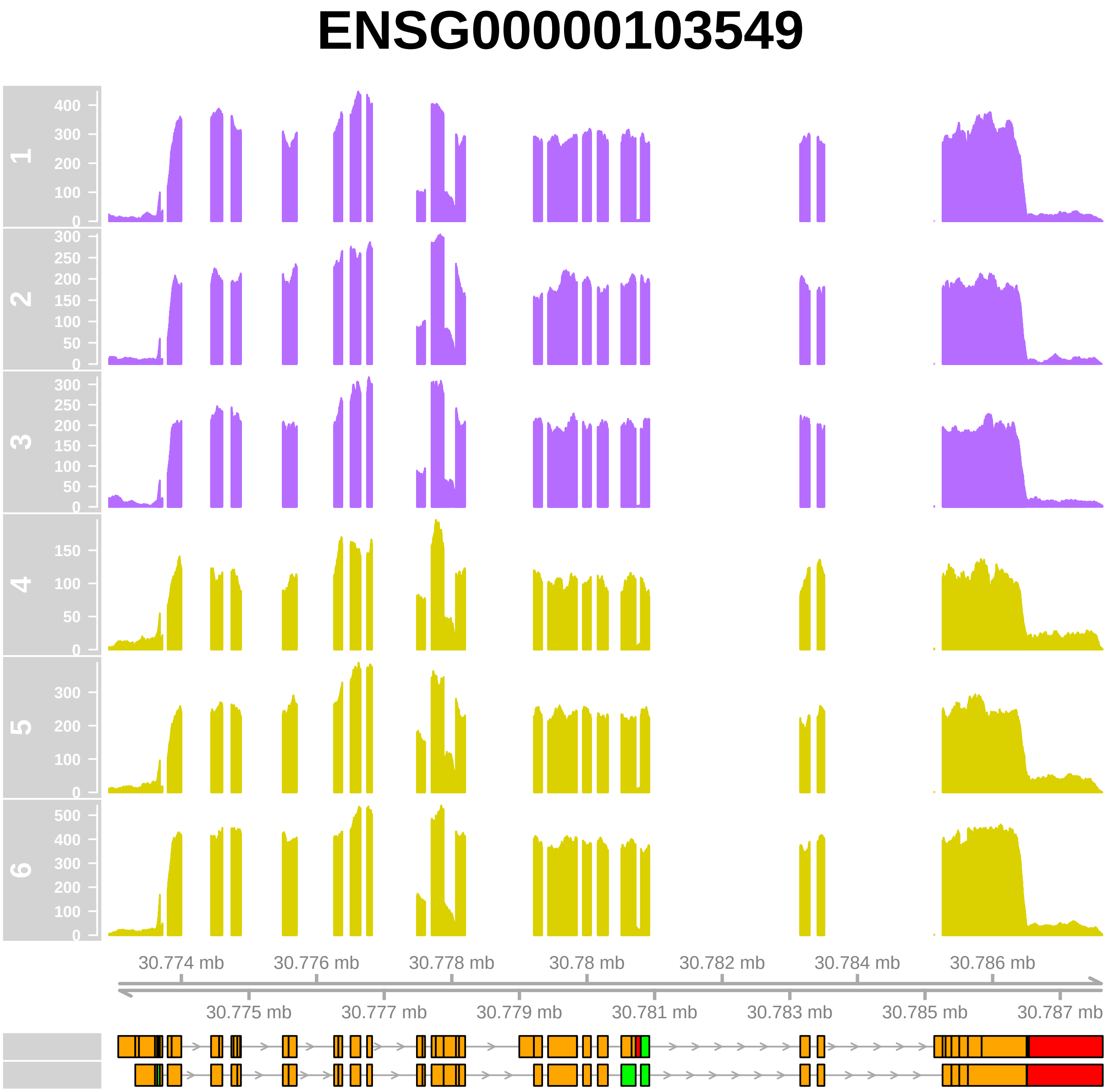

Supplementary Figure 27: Example illustration of one truly non-differential gene classified as differential after filtering (excluding all isoforms with relative abundance below 5% in both conditions) but not with the original annotation file. The first six tracks show the read coverage for each of the six samples, coloured by condition. The two tracks below the genomic coordinates illustrate the counting bins generated by DEXSeq with the original annotation file (top track) and after filtering (bottom track). Bins coloured in green are those assigned a p-value below 0.05. Bins coloured in red also have an adjusted p-value below 0.05.

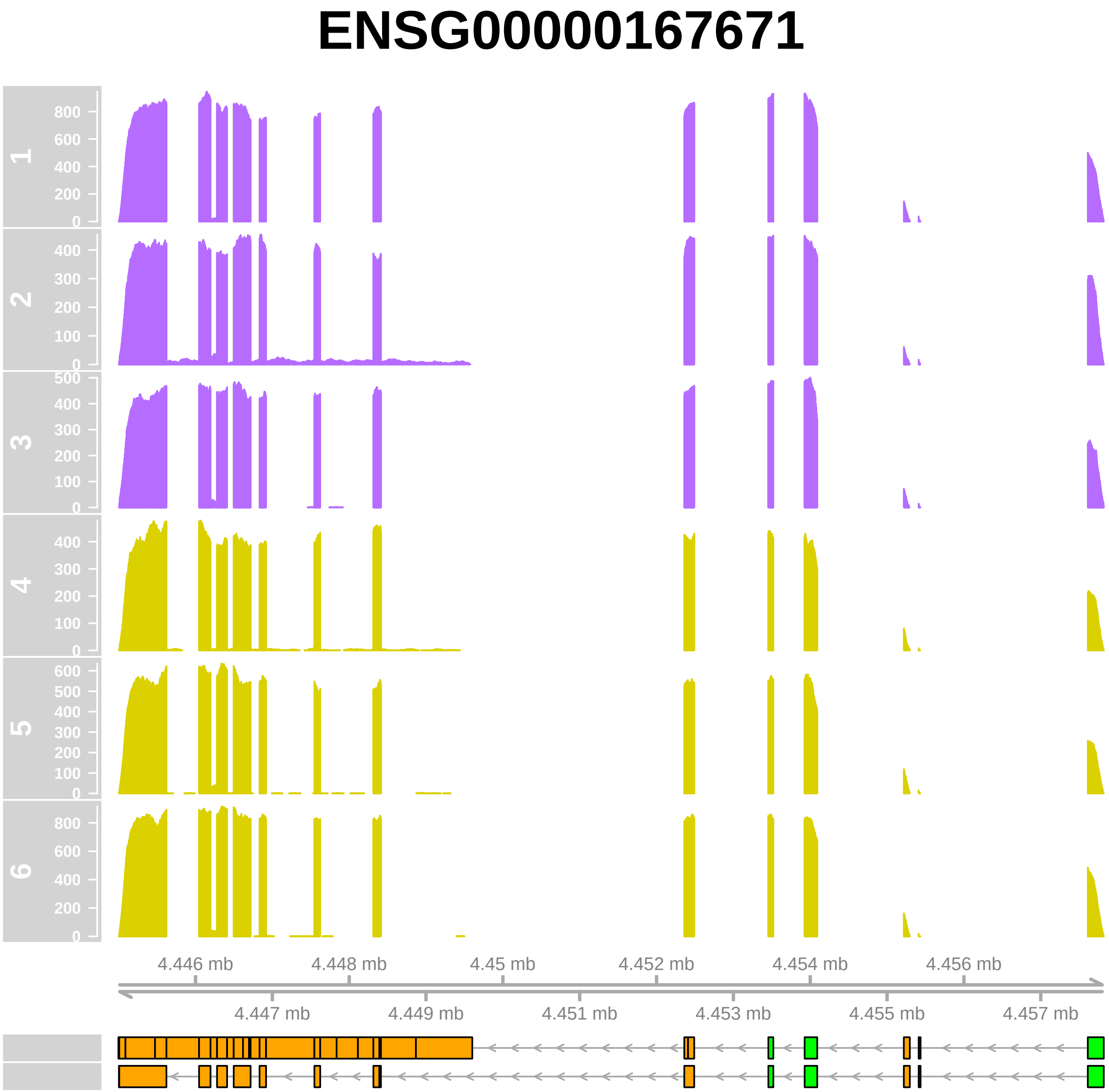

Supplementary Figure 28: Example illustration of one truly non-differential gene classified as differential after filtering (excluding all isoforms with relative abundance below 5% in both conditions) but not with the original annotation file. The first six tracks show the read coverage for each of the six samples, coloured by condition. The two tracks below the genomic coordinates illustrate the counting bins generated by DEXSeq with the original annotation file (top track) and after filtering (bottom track). Bins coloured in green are those assigned a p-value below 0.05. Bins coloured in red also have an adjusted p-value below 0.05.

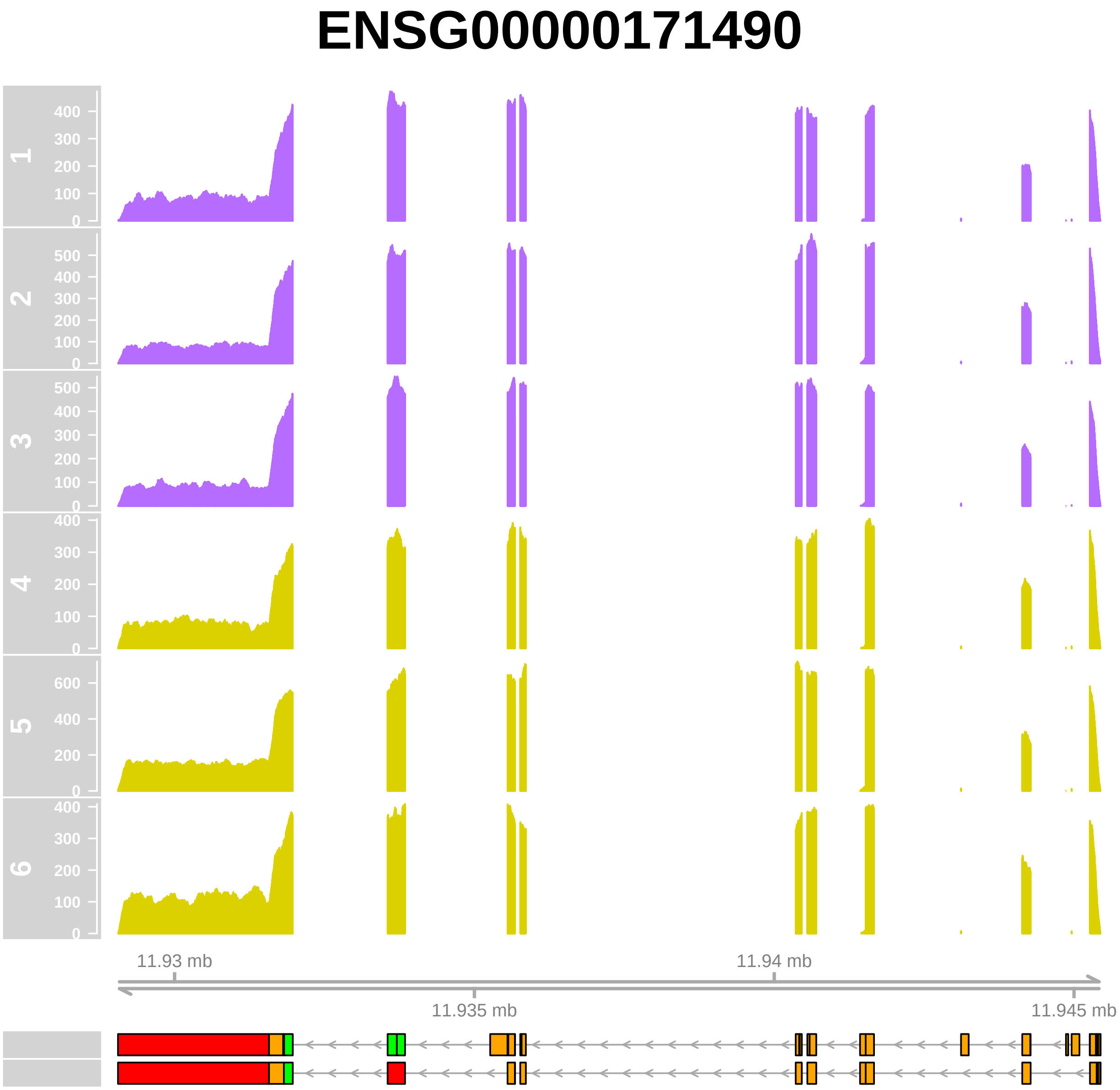

Supplementary Figure 29: Example illustration of one truly non-differential gene classified as differential after filtering (excluding all isoforms with relative abundance below 5% in both conditions) but not with the original annotation file. The first six tracks show the read coverage for each of the six samples, coloured by condition. The two tracks below the genomic coordinates illustrate the counting bins generated by DEXSeq with the original annotation file (top track) and after filtering (bottom track). Bins coloured in green are those assigned a p-value below 0.05. Bins coloured in red also have an adjusted p-value below 0.05.

ENSG00000047188

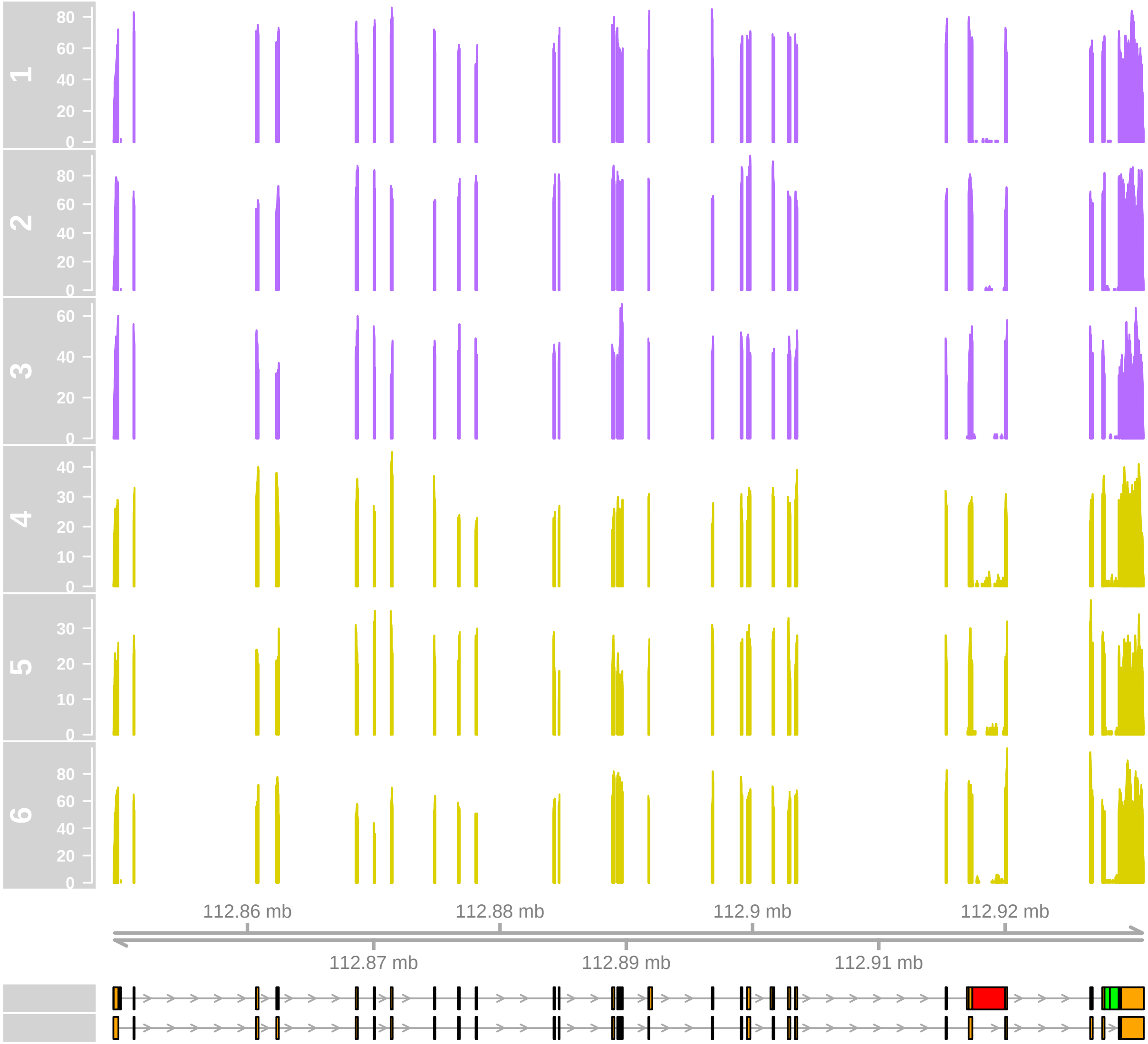

Supplementary Figure 30: Example illustration of one truly non-differential gene classified as differential with the original annotation file but not after filtering (excluding all isoforms with relative abundance below 5% in both conditions). The first six tracks show the read coverage for each of the six samples, coloured by condition. The two tracks below the genomic coordinates illustrate the counting bins generated by DEXSeq with the original annotation file (top track) and after filtering (bottom track). Bins coloured in green are those assigned a p-value below 0.05. Bins coloured in red also have an adjusted p-value below 0.05.

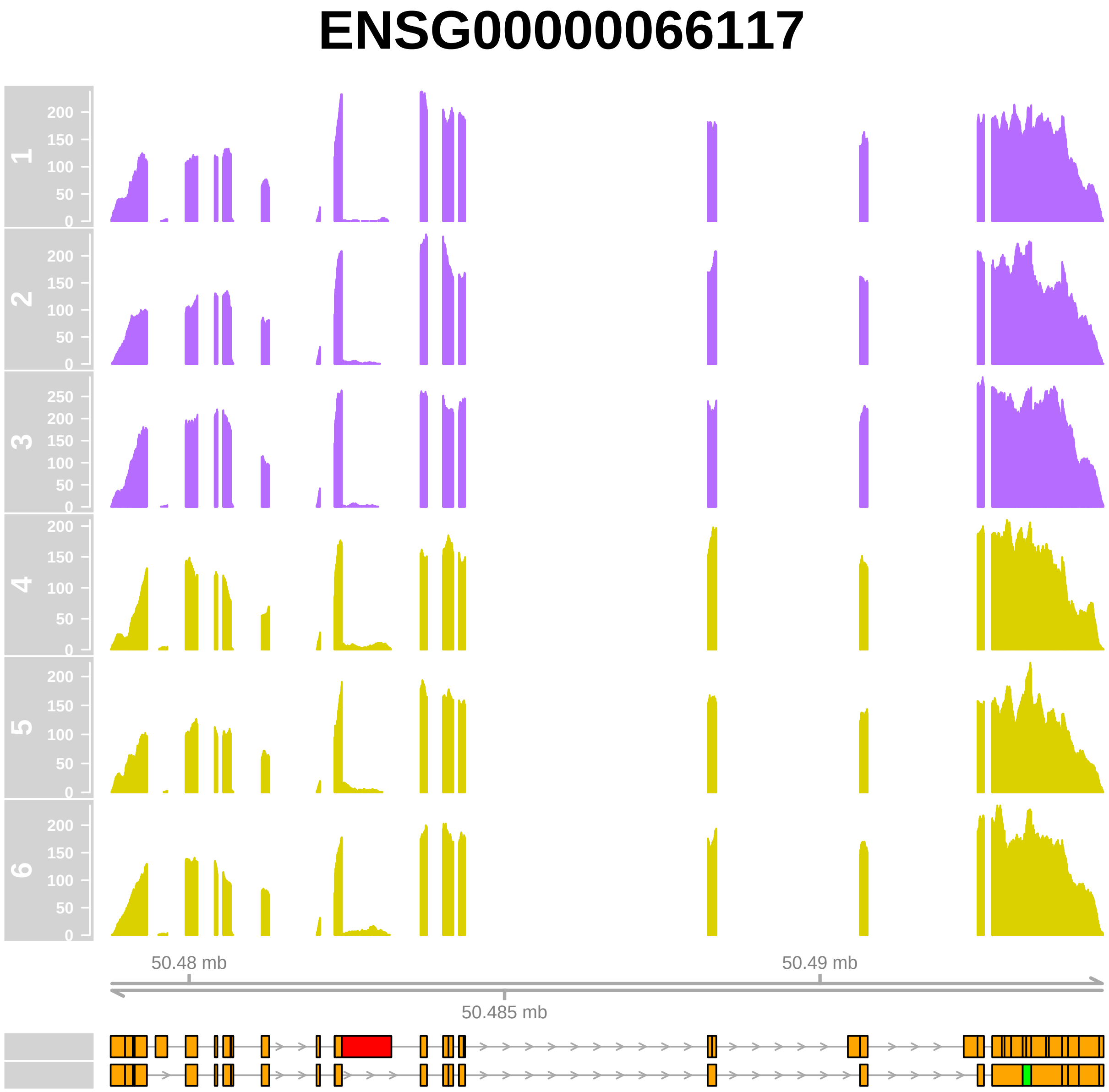

Supplementary Figure 31: Example illustration of one truly non-differential gene classified as differential with the original annotation file but not after filtering (excluding all isoforms with relative abundance below 5% in both conditions). The first six tracks show the read coverage for each of the six samples, coloured by condition. The two tracks below the genomic coordinates illustrate the counting bins generated by DEXSeq with the original annotation file (top track) and after filtering (bottom track). Bins coloured in green are those assigned a p-value below 0.05. Bins coloured in red also have an adjusted p-value below 0.05.

ENSG00000077150

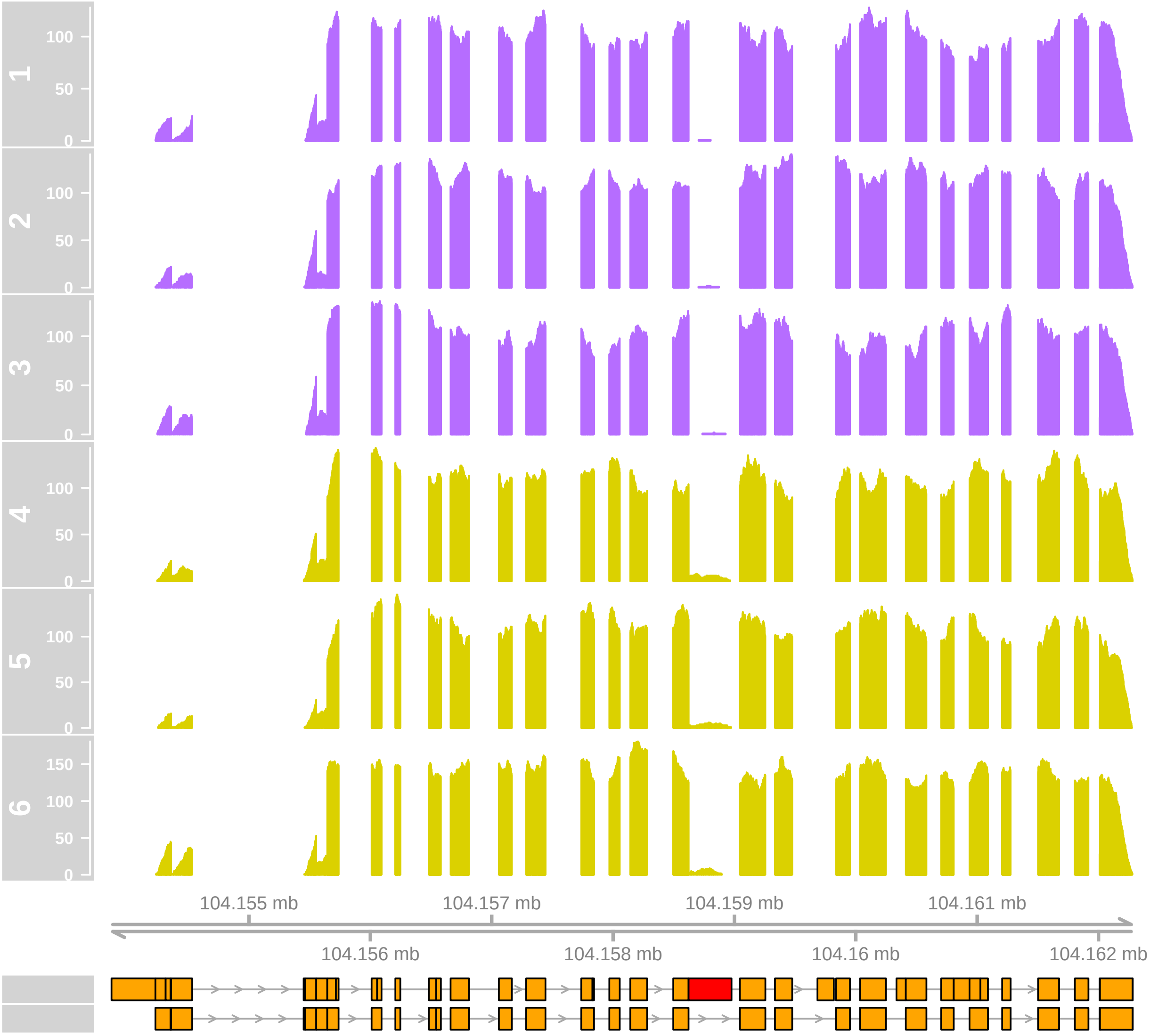

Supplementary Figure 32: Example illustration of one truly non-differential gene classified as differential with the original annotation file but not after filtering (excluding all isoforms with relative abundance below 5% in both conditions). The first six tracks show the read coverage for each of the six samples, coloured by condition. The two tracks below the genomic coordinates illustrate the counting bins generated by DEXSeq with the original annotation file (top track) and after filtering (bottom track). Bins coloured in green are those assigned a p-value below 0.05. Bins coloured in red also have an adjusted p-value below 0.05.

14.2 *kallisto*

Similarly to the analysis performed for *DEXSeq-noaggreg* in the main manuscript and in the previous subsection, we evaluated the effect of different types of filtering on the *kallisto* results (still, using DEXSeq as the inference engine). First, we used the underlying ("true") TPM values to filter out all transcripts with relative abundance (within a gene) below 5%, 10%, 15% or 25%, respectively, from the transcript fasta file generated by TopHat (which was used to generate the kallisto index for the full transcript set). From the filtered fasta files, we generated new kallisto indexes and re-quantified the remaining transcripts with kallisto, followed by differential analysis with DEXSeq. Supplementary Figure 33 compares the results obtained with the unfiltered kallisto quantifications to the ones obtained after each of the four filterings. Similarly to *DEXSeq-noaggreg*, the performance of kallisto+DEXSeq is considerably improved by the pre-filtering of the annotation catalog.

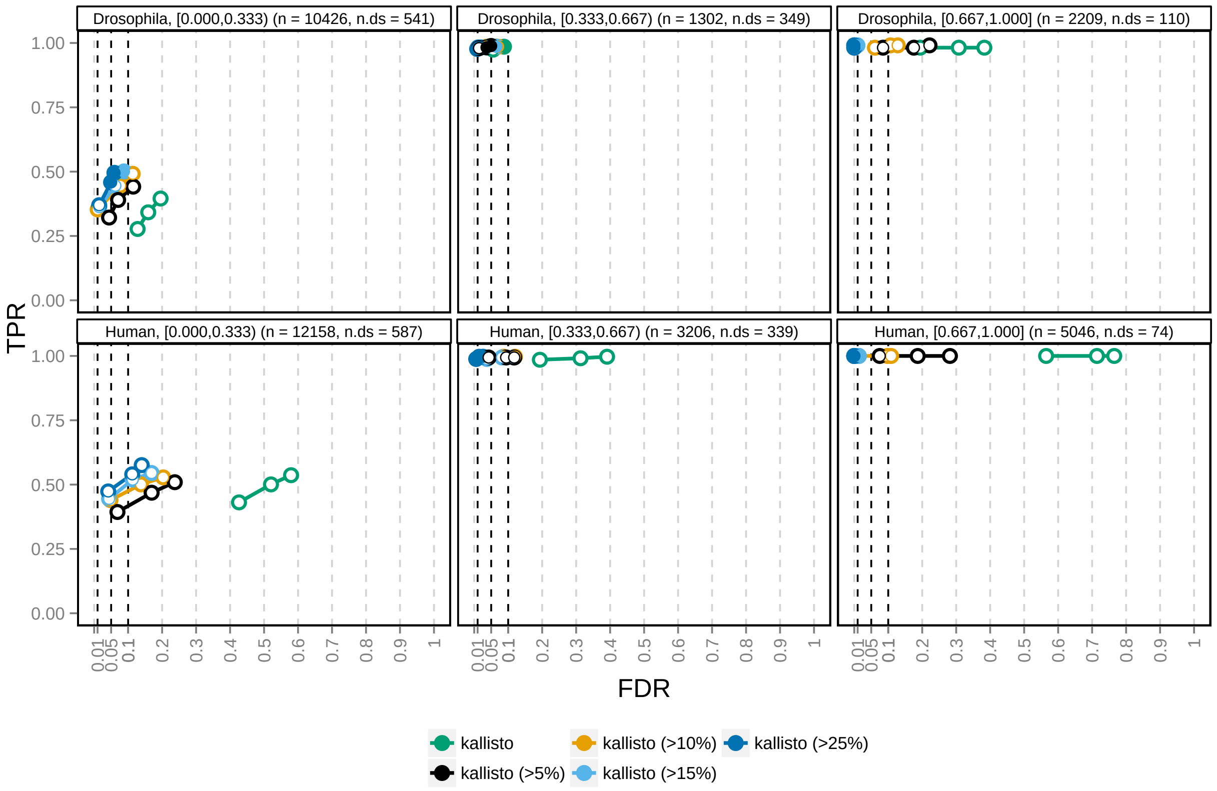

Supplementary Figure 33: Illustration of the performance of *kallisto* after various degrees of isoform-guided annotation filtering, stratified by the relative abundance difference between the two most highly abundant isoforms. Excluding lowly expressed transcripts from the annotation catalog before kallisto quantification improves the control of FDR, and leads to a slight gain of power.

In addition to the filtering based on the underlying relative abundances (Supplementary Figure 33), we evaluated two filterings based on the actual kallisto abundance estimates. First, we excluded all bins (transcripts) with an estimated relative abundance (within a gene) below 5% in all samples and applied DEXSeq to the remaining transcript counts. Second, we re-quantified the abundances of the remaining transcripts after this filtering, and applied DEXSeq to the re-estimated counts. Supplementary Figure 34 compares the different types of filtering approaches in terms of their impact on the performance of *kallisto*. While filtering based on the "true" underlying TPM values led to the largest improvement in FDR control, especially in the human simulation, filtering based on the estimated values, with or without re-estimation of

the abundances, also gave tangible performance improvements.

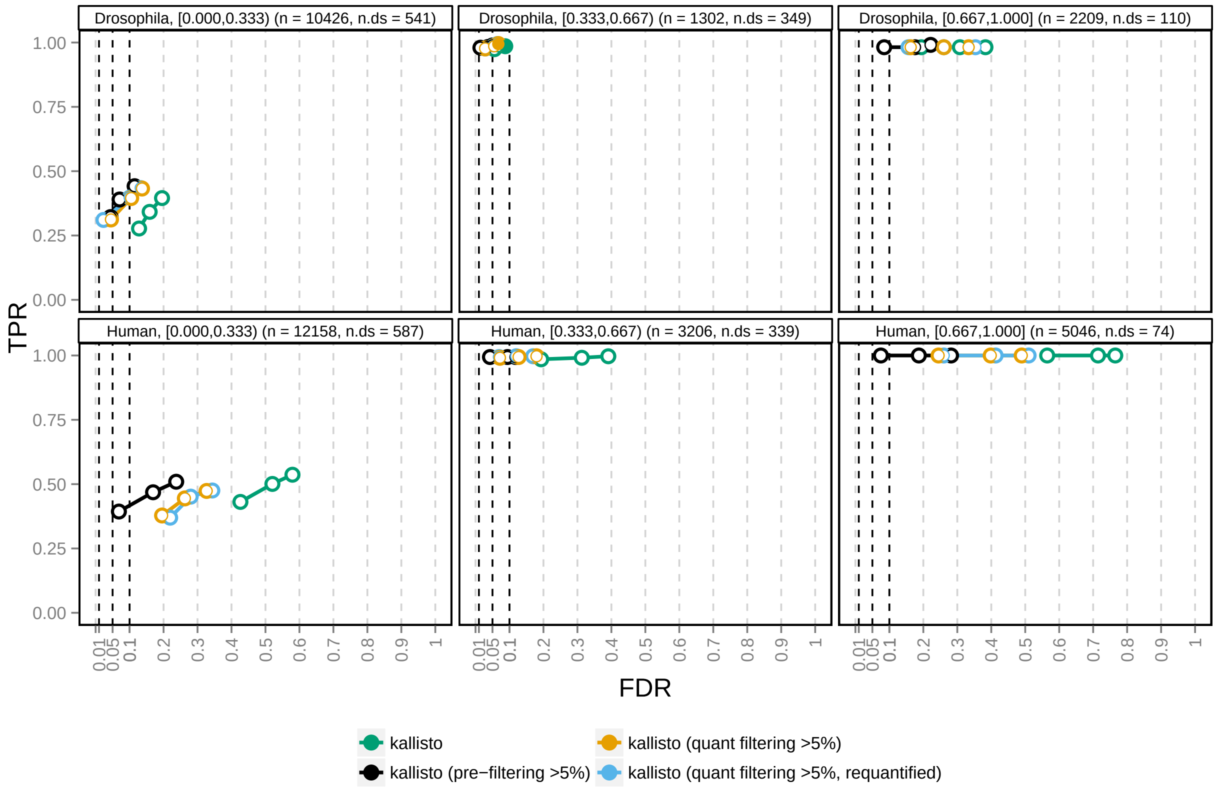

Supplementary Figure 34: Illustration of the performance of *kallisto* after different types of annotation filtering. "Pre-filtering" refers to the filtering using the underlying, "true" abundance values, while "quant filtering" refers to filtering based on the observed kallisto quantification results. Re-quantifying the abundances after filtering did not lead to a major performance improvement.

14.3 *rMATS*

In the previous sections we evaluated how isoform pre-filtering affects the performance of two counting bin definition methods (*DEXSeq-noaggreg* and *kallisto*) when used with DEXSeq. Here, we consider instead the effect of different types of filtering on the performance of *rMATS*. First, we re-ran *rMATS* using the annotation catalog obtained by excluding transcripts with a true relative abundance (within a gene) below 5% in both sample conditions, and compared to the performance based on the complete annotation catalog (cf. Figure 6 in the main text and Supplementary Figure 33 for the effect of this type of filtering on the counting methods). Second, we take advantage of the fact that *rMATS* lets the user define the null hypothesis as $H_0 : |\Delta\psi| \leq \delta$, where δ is an arbitrary non-negative value and $\Delta\psi$ is the difference in percent spliced in for a given event between two conditions. Setting $\delta > 0$ avoids small, potentially biologically irrelevant effects being called significant. We evaluated the effect of setting $\delta = 0.1$, using the full annotation catalog as the basis. Supplementary Figure 35 shows that as expected, setting $\delta > 0$ improves the FDR control quite drastically, since small, spurious changes will not be called significant. However, it also reduces the power, especially for the genes where there is a real, but small effect. We note that also for *rMATS*, the pre-filtering provides a tangible improvement compared to applying *rMATS* on the complete transcript catalog.

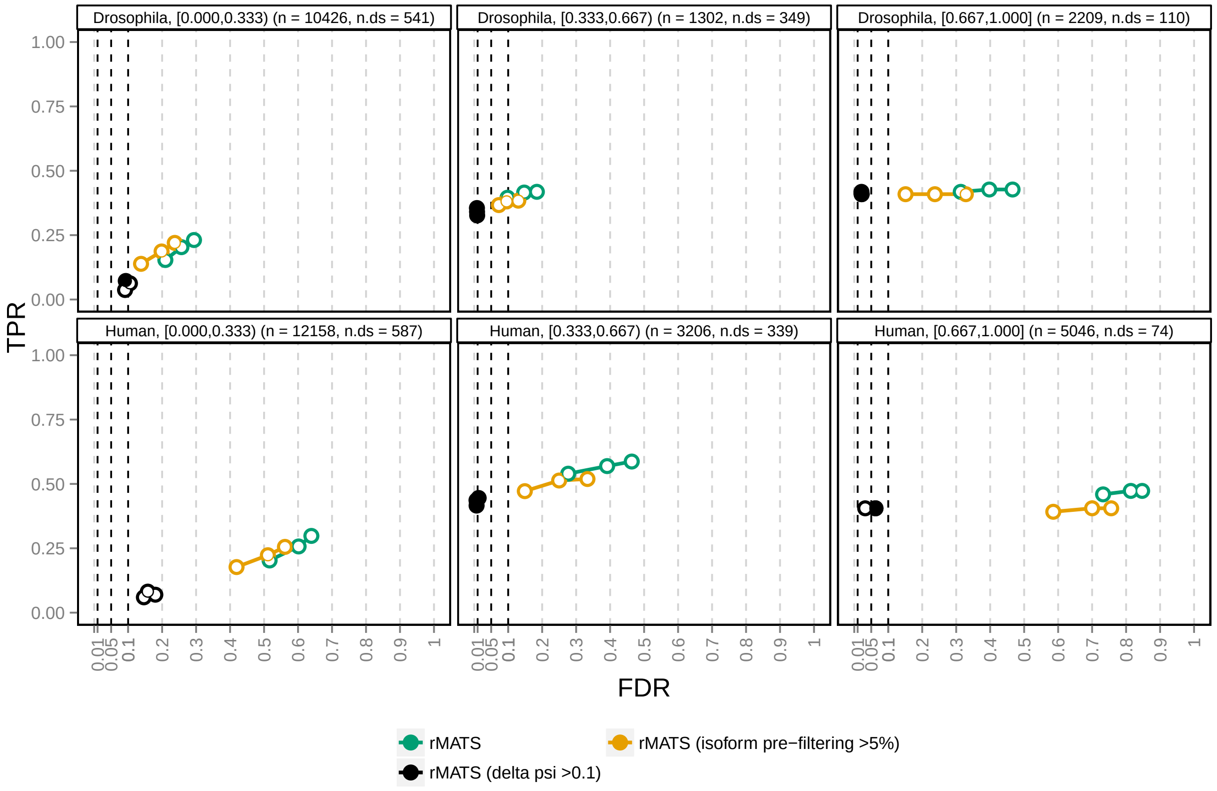

Supplementary Figure 35: Illustration of the performance of *rMATS* after different types of filtering. Isoform pre-filtering corresponds to applying *rMATS* to a filtered annotation catalog, where lowly expressed transcripts have been removed. The other evaluated modification corresponds to using $\delta = 0.1$ instead of $\delta = 0$ to define the *rMATS* null hypothesis.

References

[1] Simon Anders, Alejandro Reyes, and Wolfgang Huber. Detecting differential usage of exons from RNA-seq data. *Genome Res*, 22(10):2008–17, 2012.

[2] Nicolas Bray, Harold Pimentel, Páll Melsted, and Lior Pachter. Near-optimal RNA-Seq quantification. *arXiv preprint 1505.02710*, May 2015.

[3] Sylvain Foissac and Michael Sammeth. ASTALAVISTA: dynamic and flexible analysis of alternative splicing events in custom gene datasets. *Nucleic Acids Research*, 35(Web Server issue):W297–9, July 2007.

[4] Steffen Heber, Max Alekseyev, Sing-Hoi Sze, Haixu Tang, and Pavel A Pevzner. Splicing graphs and EST assembly problem. *Bioinformatics*, 18 Suppl 1:S181–S188, 2002.

[5] Wolfgang Huber, Vincent J Carey, Robert Gentleman, Simon Anders, Marc Carlson, Benilton S Carvalho, Hector Corrada Bravo, Sean Davis, Laurent Gatto, Thomas Girke, Raphael Gottardo, Florian Hahne, Kasper D Hansen, Rafael A Irizarry, Michael Lawrence, Michael I Love, James Macdonald, Valerie Obenchain, Andrzej K Oleś, Hervé Pagès, Alejandro Reyes, Paul Shannon, Gordon K Smyth, Dan Tenenbaum, Levi Waldron, and Martin Morgan. Orchestrating high-throughput genomic analysis with Bioconductor. *Nat Methods*, 12(2):115–21, 2015.

[6] Yarden Katz, Eric T Wang, Edoardo M Airoldi, and Christopher B Burge. Analysis and design of RNA sequencing experiments for identifying isoform regulation. *Nat Methods*, 7(12):1009–15, 2010.

[7] Yang Liao, Gordon K Smyth, and Wei Shi. The Subread aligner: fast, accurate and scalable read mapping by seed-and-vote. *Nucleic Acids Res*, 41(10):e108, May 2013.

[8] Yang Liao, Gordon K Smyth, and Wei Shi. featureCounts: an efficient general purpose program for assigning sequence reads to genomic features. *Bioinformatics*, 30(7):923–30, April 2014.

[9] David Rossell, Camille Stephan-Otto Attolini, Manuel Kroiss, and Almond Stöcker. Quantifying Alternative Splicing From Paired-End Rna-Sequencing Data. *Ann Appl Stat*, 8(1):309–30, 2014.

[10] Michael Sammeth. Complete alternative splicing events are bubbles in splicing graphs. *Journal of Computational Biology*, 16(8):1117–1140, 2009.

[11] Michael Sammeth, Sylvain Foissac, and Roderic Guigó. A general definition and nomenclature for alternative splicing events. *PLoS Computational Biology*, 4(8), 2008.

[12] Charlotte Soneson and Mauro Delorenzi. A comparison of methods for differential expression analysis of RNA-seq data. *BMC Bioinformatics*, 14(1):91, January 2013.

[13] C W Sugnet, W J Kent, M Ares, and D Haussler. Transcriptome and genome conservation of alternative splicing events in humans and mice. *Pacific Symposium on Biocomputing*, pages 66–77, 2004.
